# Supplementary material for: A Physiologically Based Pharmacokinetic Model of Ketoconazole and Its Metabolites as Drug–Drug Interaction Perpetrators
Source: Pharmaceutics. 2023 Feb 17;15(2):679. doi: 10.3390/pharmaceutics15020679 (PMC9965990; doi:10.3390/pharmaceutics15020679)
Supplement: Supplementary file 1 [file pharmaceutics-15-00679-s001.zip › pharmaceutics-2148764-supplementary.pdf]

Pharmaceutics

A Physiologically based Pharmacokinetic Model of Ketoconazole and its Metabolites as Drug-Drug Interaction Perpetrators

Supplementary Material

Fatima Zahra Marok[1], Jan-Georg Wojtyniak[1,2], Laura Maria Fuhr[1], Matthias Schwab[2,3,4], Johanna Weiss[5,6], Walter Emil Haefeli[5,6], Thorsten Lehr[1]

[1]Clinical Pharmacy, Saarland University, Saarbruecken, Germany
[2]Dr. Margarete Fischer-Bosch-Institut of Clinical Pharmacology, Stuttgart, Germany
[3]Departments of Clinical Pharmacology, and of Biochemistry and Pharmacy, University Hospital Tuebingen, Germany
[4]Cluster of excellence iFIT (EXC2180) “Image-Guided and Functionally Instructed Tumor Therapies”, University Tuebingen, Germany
[5]Department of Clinical Pharmacology and Pharmacoepidemiology, University of Heidelberg, Germany
[6]German Center for Infection Research (DZIF), Heidelberg Partner Site, Germany

Funding:

M.S. was supported by the Robert Bosch Stiftung (Stuttgart, Germany), the European Commission Horizon 2020 UPGx grant 668353, a grant from the German Federal Ministry of Education and Research (BMBF 031L0188D), and the Deutsche Forschungsgemeinschaft (DFG, German Research Foundation) under Germany’s Excellence Strategy—EXC 2180—390900677. T.L. was supported by the the project “Open-source modeling framework for automated quality control and management of complex life science system models” (OSMOSES) funded by the German Federal Ministry of Education and Research (BMBF, grant ID:031L0161C).

Conflict of Interest:

Since January 2020 Jan-Georg Wojtyniak is an employe of Boehringer Ingelheim Pharma GmbH and Co. KG. All other authors declare no conflict of interest.

Corresponding Author:

Prof. Dr. Thorsten Lehr

Clinical Pharmacy, Saarland University

Campus C5 3, 66123 Saarbrücken, Germany

Phone: +49 681 302 70255

Email: thorsten.lehr@mx.uni-saarland.de

Contents

S1 PBPK Model Building **2**
S1.1 System-dependent parameters . . . 2
S1.2 Mathematical implementation of drug interactions . . . 3
S1.2.1 Drug-food interactions (DFIs) . . . 3
S1.2.2 Drug-drug interactions (DDIs) . . . 3
S1.3 Physiologically based pharmacokinetic (PBPK) model evaluation . . . 4
S1.4 Sensitivity analysis . . . 4
S1.5 Ketoconazole – Clinical studies . . . 5
S1.6 Ketoconazole – Drug-dependent parameters . . . 7

S2 Ketoconazole – PBPK model evaluation **9**
S2.1 Plasma concentration-time profiles (Linear) . . . 9
S2.2 Plasma concentration-time profiles (Semilogarhitmic) . . . 16
S2.3 Predicted compared to observed concentrations goodness-of-fit plots . . . 23
S2.4 AUC_{last} and C_{max} goodness-of-fit plots . . . 23
S2.5 Mean relative deviation of plasma concentration predictions . . . 24
S2.6 Geometric mean fold error of predicted AUC_{last} and C_{max} values . . . 26
S2.7 Ketoconazole – DFI model evaluation . . . 28
S2.7.1 Plasma concentration-time profiles (Linear) . . . 28
S2.7.2 Plasma concentration-time profiles (Semilogarhitmic) . . . 29
S2.7.3 DFI AUC_{last} and DFI C_{max} goodness-of-fit plots . . . 30
S2.7.4 Geometric mean fold error of predicted AUC_{last} and C_{max} values . . . 31
S2.8 Sensitivity Analyses . . . 32

S3 Ketoconazole – DDI Modeling **34**
S3.1 Ketoconazole – Clinical studies . . . 34
S3.2 Ketoconazole – Drug-dependent parameters . . . 36
S3.3 Ketoconazole – DDI model evaluation . . . 36
S3.3.1 Plasma concentration-time profiles (Linear) . . . 36
S3.3.2 Plasma concentration-time profiles (Semilogarhitmic) . . . 42
S3.3.3 DDI AUC_{last} and DDI C_{max} goodness-of-fit plots . . . 48
S3.3.4 Geometric mean fold error of predicted DDI AUC_{last} and DDI C_{max} ratios . . . 49

References **53**

S1 PBPK Model Building

S1.1 System-dependent parameters

Table S1.1: System-dependent parameter

Protein	Reference concentration			Localization	Half-life	
	Mean[a]	GSD[b]	Relative expression[c]		liver [h]	intestine [h]
AADAC	[d]1.00 [1]	[e]1.40	RT-PCR [2]	Intracellular	36	23
CYP3A4	4.32 [3]	1.18 liver [4]<br>1.46 int. [4]	RT-PCR [5]	Intracellular	36 [6]	23 [7]
FMO3	[d]1.00	1.00 [1]	RT-PCR[2]	Intracellular	-	23
UGT1A4	[f]1.32 [8]	1.07[4]	RT-PCR [2]	Intracellular	36	23
P-gp	[h]1.41	1.60 [9]	RT-PCR [10]	Apical (Efflux)	36	23

AADAC: arylacetamide deacetylase, CYP: cytochrome P450, FMO: flavin-containing monooxygenase, P-gp: P-glycoprotein, RT-PCR: reverse transcription-polymerase chain reaction measured expression profile, UGT: UDP-glucuronosyltransferase.

[a] μmol protein/l in the tissue of highest expression

[b] geometric standard deviation of the reference concentration

[c] in the different organs (PK-Sim® expression database profile)

[d] if no information was available, the mean reference concentration was set to 1.00 µmol/l and the catalytic rate constant was optimized according to [1]

[e] if no information was available, a moderate variability of 35% CV was assumed (= 1.40 GSD)

[f] calculated from protein per mg microsomal protein x 40 mg microsomal protein per g liver [11]

[g] calculated from transporter per mg membrane protein x 26.2 mg human kidney microsomal protein per g kidney [11]

[h] optimized

S1.2 Mathematical implementation of drug interactions

S1.2.1 Drug-food interactions (DFIs)

DFIs were implemented by extending the gastric emptying time (GET). Extended GET was assumed for clincial studies with (i) reported food intake, (ii) a delayed observed time of maximum concentration (T_{max}) of more than two hours, (iii) multiple dose asministrations within a day and (iv) doses above 600 mg.

S1.2.2 Drug-drug interactions (DDIs)

The simulated DDIs included competitive inhibition of cytochrome P450 (CYP3A4) and P-glycoprotein (P-gp).

Equation: Competitive inhibition

$$K_{M,app} = K_M * (1 + \frac{[I]}{K_i}) \tag{S1}$$

$$v = \frac{v_{max} * [S]}{K_{M,app} + [S]} = \frac{k_{cat} * [E] * [S]}{K_{M,app} + [S]} \tag{S2}$$

$K_{M,app}$ = Michaelis-Menten constant in the presence of inhibitor
K_M = Michaelis-Menten constant
$[I]$ = free inhibitor concentration
K_i = dissociation constant of the inhibitor-protein complex
v = reaction velocity
$[S]$ = free substrate concentration
k_{cat} = catalytic/transport rate constant
$[E]$ = protein concentration

S1.3 Physiologically based pharmacokinetic (PBPK) model evaluation

The model performances were evaluated by illustrating predicted and observed plasma concentration-time profiles and goodness-of-fit plots. Furthermore, the models were evaluated by comparing predicted to observed area under the plasma concentration-time curve from the time of drug administration to the last concentration measuremente (AUC_{last}) and maximum plasma concentration (C_{max}) values.

As quantitative performance measures, a mean relative deviation (MRD) was calculated for all profiles from their respective predicted and observed plasma concentrations. Furthermore, the geometric mean fold error (GMFE) of the AUC_{last} and C_{max} were calculated.

S1.4 Sensitivity analysis

Sensitivity of the final models to single parameters (local sensitivity analysis) was calculated as relative change of the area under the plasma concentration-time curve extrapolated to infinity (AUC_{inf}). The analysis was carried out using a relative perturbation of 1000% (variation range 10.0, maximum number of 9 steps). Parameters were included into the analysis if they have been optimized, if they are associated with optimized parameters or if they might have a strong impact due to calculation methods used in the model. Sensitivity to a parameter changes was calculated according to Eq. (S3):

Equation: Sensitivity analysis

$$S = \frac{\Delta AUC_{inf}}{\Delta p} * \frac{p}{AUC_{inf}} \quad \text{(S3)}$$

S = sensitivity of the AUC_{inf} to the examined model parameter
ΔAUC_{inf} = change of the AUC_{inf}
AUC_{inf} = simulated, AUC_{inf} with the original parameter value
Δp = change of the examined parameter value
p = original parameter value

A sensitivity of + 1.0 signifies that a 10% increase of the examined parameter value causes a 10% increase of the simulated AUC.

S1.5 Ketoconazole – Clinical studies

Table S1.2: Clinical study data used for ketoconazole model development

Dose [mg]	Route	N	Age [years]	Weight [kg]	Height [cm]	BMI [kg/m²]	Females [%]	Dataset	Reference
200	sol s.d	12	-	-	-	-	-	training	Heel 1982 [12]
200	sol s.d	12	20 (18-25)	76.4 (61.2-95.3)	180 (167.6-188)	-	0	test	Huang 1986a [13]
200	sol s.d	23	20 (18-25)	76.4 (61.2-95.3)	180 (167.6-188)	-	0	test	Huang 1986b [13]
400	sol s.d	12	20 (18-25)	76.4 (61.2-95.3)	180 (167.6-188)	-	0	test	Huang 1986b [13]
800	sol s.d	12	20 (18-25)	76.4 (61.2-95.3)	180 (167.6-188)	-	0	training	Huang 1986b [13]
100	tab s.d	12	-	-	-	-	-	test	Heel 1982 [12]
200	tab s.d	9	(22-41)	-	-	-	33.34	test	Chin 1995 [14]
200	tab s.d	8	25 (21-46)	-	-	-	0	test	Daneshmend 1983 [15]
200	tab s.d	8	23 (20-31)	64 (50-75)	-	-	62.5	test	Daneshmend 1984a [16]
200	tab s.d	8	23 (20-31)	64 (50-75)	-	-	62.5	training	Daneshmend 1984b [16]
200	tab s.d	23	-	-	-	-	-	test	FDA 1998b [17]
200	tab s.d	39	-	-	-	-	-	test	FDA 1998a [17]
200	tab s.d	39	-	-	-	-	-	training	FDA 1998a [17]
200	tab s.d	12	-	-	-	-	-	test	Heel 1982 [12]
200	tab s.d	23	20 (18-25)	76.4 (61.2-95.3)	180 (167.6-188)	-	0	test	Huang 1986a [13]
200	tab s.d	12	30 (24-36)	78.8	180.7	-	0	test	Knupp 1993 [18]
200	tab s.d	10	24 (22-26)	62 (55-70)	-	-	50	test	Mannistoe 1982 [19]
400	tab s.d	12	(23-29)	(59-78)	-	-	0	test	Sadeghina 2005b [20]
400	tab s.d	12	(23-29)	(59-78)	-	-	0	test	Sadeghina 2005a [20]
200	tab s.d	24	23.2 (18-45)	-	-	22.5 (20-24)	0	test	Solomon 2007b [21]
200	tab s.d	24	23.2 (18-45)	-	-	22.5 (20-24)	0	test	Solomon 2007a [21]
200	tab s.d	3	(28-42)	-	-	-	-	test	Van der Meer 1980 [22]
200	tab s.d	18	-	-	-	-	-	test	Yuen 1999a [23]
200	tab s.d	18	-	-	-	-	-	test	Yuen 1999b [23]
400	tab s.d	8	23 (20-31)	64 (50-75)	-	-	62.5	test	Daneshmend 1984a [16]
400	tab s.d	8	23 (20-31)	64 (50-75)	-	-	62.5	test	Daneshmend 1984b [16]
400	tab s.d	12	-	-	-	-	-	test	Heel 1982 [12]
400	tab s.d	12	23 (19-41)	77.4 (64.2-99.8)	175.8 (163.2-185.4)	-	0	test	Polk 1999 [24]
400	tab s.d	6	(18-30)	-	-	-	0	test	Piscitelli 1991 [25]
400	cap s.d	12	33.7 (22-55)	-	-	22.56 (20.34-28.04)	75	test	Sriwiriyajan 2007 [26]
400	tab s.d	12	27.34 (20-48)	74.44 (57.5-100)	173.33 (162-180)	25.27 (21.9-29.9)	0	test	Weiss 2022 [27]
600	tab s.d	8	23 (20-31)	64 (50-75)	-	-	62.5	training	Daneshmend 1984a [16]
600	tab s.d	8	23 (20-31)	64 (50-75)	-	-	62.5	test	Daneshmend 1984b [16]
800	tab s.d	8	23 (20-31)	64 (50-75)	-	-	62.5	test	Daneshmend 1984a [16]
800	tab s.d	8	23 (20-31)	64 (50-75)	-	-	62.5	training	Daneshmend 1984b [16]
200	tab m.d	24	26.6 (18-39)	73.5 (53.8-98.8)	-	23.8 (18-28)	41.67	test	Boyce 2012b [28]

(Continued on next page...)

Table S1.2: Clinical study data used for Ketoconazole model development *(continued)*

Dose [mg]	Route	N	Age [years]	Weight [kg]	Height [cm]	BMI [kg/m²]	Females [%]	Dataset	Reference
200	tab m.d	24	26.6 (18-39)	73.5 (53.8-98.8)	-	23.8 (18-28)	41.67	test	Boyce 2012a [28]
200	tab m.d	8	25 (21-46)	-	-	-	0	test	Daneshmend 1983 [15]
200	tab m.d	15	36 (22-43)	74.7 (50.1-95)	168.8 (154-179)	26 (21.1-30.3)	19	test	Patel 2011 [29]
200	tab m.d	21	-	-	-	-	-	test	Tiseo 2002 [30]
200	tab m.d	15	-	-	-	-	-	training	Wire 2007 [31]
200	tab m.d	8	(18-38)	-	-	-	-	test	Greenblatt 1998 Control [32]
800	tab m.d	2	-	-	-	-	-	test	Craven 1983 [33]
1200	tab m.d	2	-	-	-	-	-	test	Craven 1983 [33]

BMI: body mass index, cap: capsule, m.d: multiple dose, N: number of individuals studied, Route: route of administration, tab: tablet, s.d: single dose, sol: solution
-: no data available. Values are means with ranges, if available.
* median

S1.6 Ketoconazole – Drug-dependent parameters

Table S1.3: Drug-dependent parameters of the ketoconazole PBPK model

Parameter	Unit	Value	Source	Literature	Reference
Ketoconazole					
MW	g/mol	531.43	lit.	531.43	[35]
logP	-	2.52	lit.	2.73	[36]
f_u	%	1	lit.	1	[12]
pka (base)	-	2.94, 6.51	lit.	2.94, 6.51	[35]
Solubility (pH)	mg/ml	$2.03 \cdot 10^4$ (1.2), $4.3 \cdot 10^4$ (3), 7.00 (6.8). 5.40 (7), 6.00 (7.5)	lit.	$2.03 \cdot 10^4$ (1.2), $4.3 \cdot 10^4$ (3), 7.00 (6.8). 5.40 (7), 6.00 (7.5)	[37]
Density	g/cm^3	1.40	lit.	1.40	[38]
Aqueous diffusion coefficient	dm^2/min	$3.75 \cdot 10^{-7}$	opt.	*$2.56 \cdot 10^{-6}$	[4]
Spec. intest. perm. fasted	cm/min	$1.56 \cdot 10^{-5}$	opt.	*$4.28 \cdot 10^{-6}$	[4]
Spec. intest. perm. fed	cm/min	$9.95 \cdot 10^{-6}$	opt.	*$4.28 \cdot 10^{-6}$	[4]
GET fasted	min	15	lit.	15	[4]
GET fed	min	45	asm.	45–120	[39]
Cellular permeabilities	-	PK-Sim	-	-	[4]
Partition coefficient	-	Berez.	-	-	[40]
GFR fraction	-	1	asm.	-	-
EHC fraction	-	1	asm.	-	-
AADAC K_M	nmol/l	1880	lit.	1880	[41]
AADAC k_{cat}	1/min	0.87	opt.	-	-
CYP3A4 K_M	nmol/l	8.46	asm.	-	[27]
CYP3A4 k_{cat}	1/min	0.10	opt.	-	-
UGT1A4 K_M	nmol/l	7000	asm.	-	[42]
UGT1A4 k_{cat}	1/min	0.31	opt.	-	-
P-gp K_M	nmol/l	35	asm.	-	[27]
P-gp k_{cat}	1/min	0.33	opt.	-	-
CYP3A4 K_i	nmol/l	8.46	lit.	[a]8.46	[27]
P-gp K_i	nmol/l	35	lit.	[a]35	[27]
Particle dissolution [b]r (Bin1)	nm	11.75	calc.	[b]11.75	[43]
Particle dissolution [c]r (Bin2)	nm	111.06	calc.	[c]111.06	[43]
Particle dissolution [d]r (Bin3)	nm	205.46	calc.	[d]205.46	[43]
N-Deacetylketoconazole					
MW	g/mol	489.40	lit.	489.40	[44]
logP	-	3.75	lit.	4.58	[44]
f_u	%	1	asm.	[e]1	[12]
pka (base)	-	0.20, 6.42, 8.90	lit.	0.20, 6.42, 8.90	[44]
Solubility (pH)	mg/ml	1240 (6.5)	lit.	1240 (6.5)	[44]
Cellular permeabilities	-	Ch.d.S.	-	-	[45]
Partition coefficient	-	R&R	-	-	[46]
GFR fraction	-	1	asm.	-	-
EHC fraction	-	1	asm.	-	-
FMO3 K_M	nmol/l	1170	lit.	1170	[47]
FMO3 k_{cat}	1/min	378.65	opt.	-	-
CYP3A4 K_i	nmol/l	22	lit.	[a]52	[27]
P-gp K_i	nmol/l	119	lit.	[a]119	[27]

(Continued on next page...)

Table S1.3: Drug-dependent parameters of the ketoconazole PBPK model *(continued)*

Parameter	Unit	Value	Source	Literature	Reference
N-Deacetyl-N-hydroxyketoconazole					
MW	g/mol	505.40	lit.	505.40	[48]
logP	-	4.20	lit.	4.20	[48]
f_u	%	1	asm.	[e]1	[12]
pka (base)	-	3.42, 6.42	lit.	3.42, 6.42	[48]
Solubility (pH)	mg/ml	4400 (6.5)	lit.	4400 (6.5)	[48]
Organ permeability	cm/min	0	asm.	*0.05	[4]
Cellular permeabilities	-	Ch.d.S.	-	-	[45]
Partition coefficient	-	Berez.	-	-	[40]
GFR fraction	-	1	asm.	-	-
EHC fraction	-	1	asm.	-	-
FMO3 Cl	l/µmol/min	0.09	opt.	-	-
CYP3A4 K_i	nmol/l	22	asm.	[a,f]52	[27]
P-gp K_i	nmol/l	119	asm.	[a,f]119	[27]

AADAC: arylacetamide deacetylase, asm.: assumed, Berez.: Berezhkovskiy calculation method, calc.: calculated, Ch.d.S.: charge dependent Schmitt calculation method, Cl: first order clearance rate constant,CYP3A4: cytochrome P450 3A4, EHC: enterohepatic circulation, FMO3: flavin-containing monooxygenase 3, f_u: fraction unbound, GET: gastric emptying time, GFR: glomerular filtration rate, intest.: intestinal, k_{cat}: catalytic/transport rate constant, K_i: concentration for half-maximal inhibition, K_M: Michaelis-Menten constant, lit.: literature, logP: lipophilicity, MW: molecular weight, opt.: optimized, P-gp: P-glycoprotein, perm.: permeability, pka: acid dissociation constant, PK-Sim: PK-Sim® standard calculation method , r: particle radii used for particle dissolution, R&R: Rodgers and Rowland, spec.: specific, tab: tablet, UGT1A4: uridine diphosphate glucuronosyltransferase 1A4,

* calculated by the software

[a] *in-vitro* values calculated from respective IC50 values. Inhibitions were implemented as competitive inhibitions.

[b] respective particle radii for 99.0025% of dose (calculated according to Dallmann et al. [34])

[c] respective particle radii for 0.895% of dose (calculated according to Dallmann et al. [34])

[d] respective particle radii for 0.1025% of dose (calculated according to Dallmann et al. [34])

[e] assumed from value for ketoconazole, as no data available

[f] assumed from value for N-deacetylketoconazole, as no data available

-: no data available.

S2 Ketoconazole – PBPK model evaluation

S2.1 Plasma concentration-time profiles (Linear)

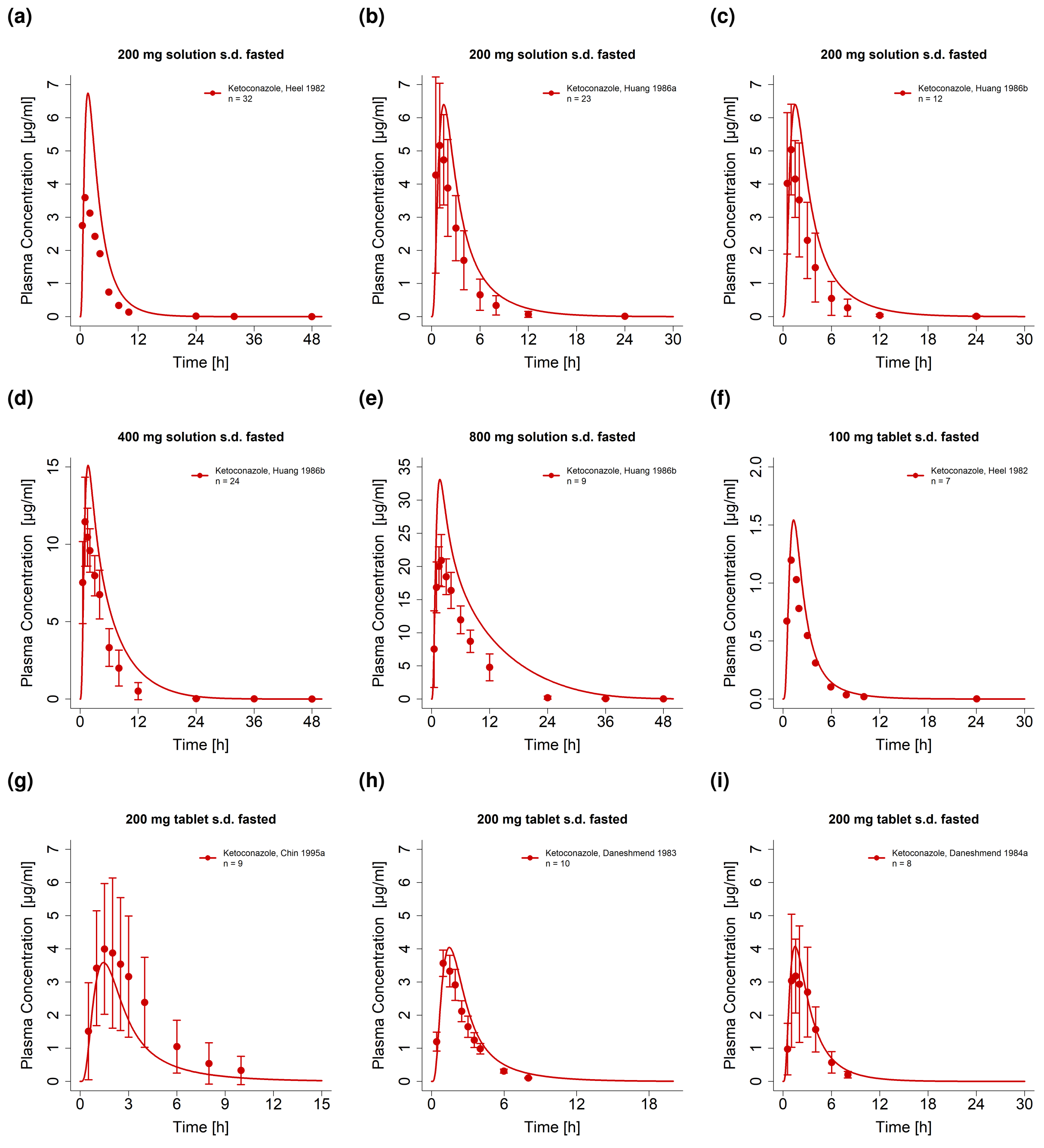

Figure S2.1: Ketoconazole plasma concentration-time profiles. Model predictions are shown as lines, observed data as dots (arithmetic mean ± SD). n: number of individuals studied, s.d: single dose

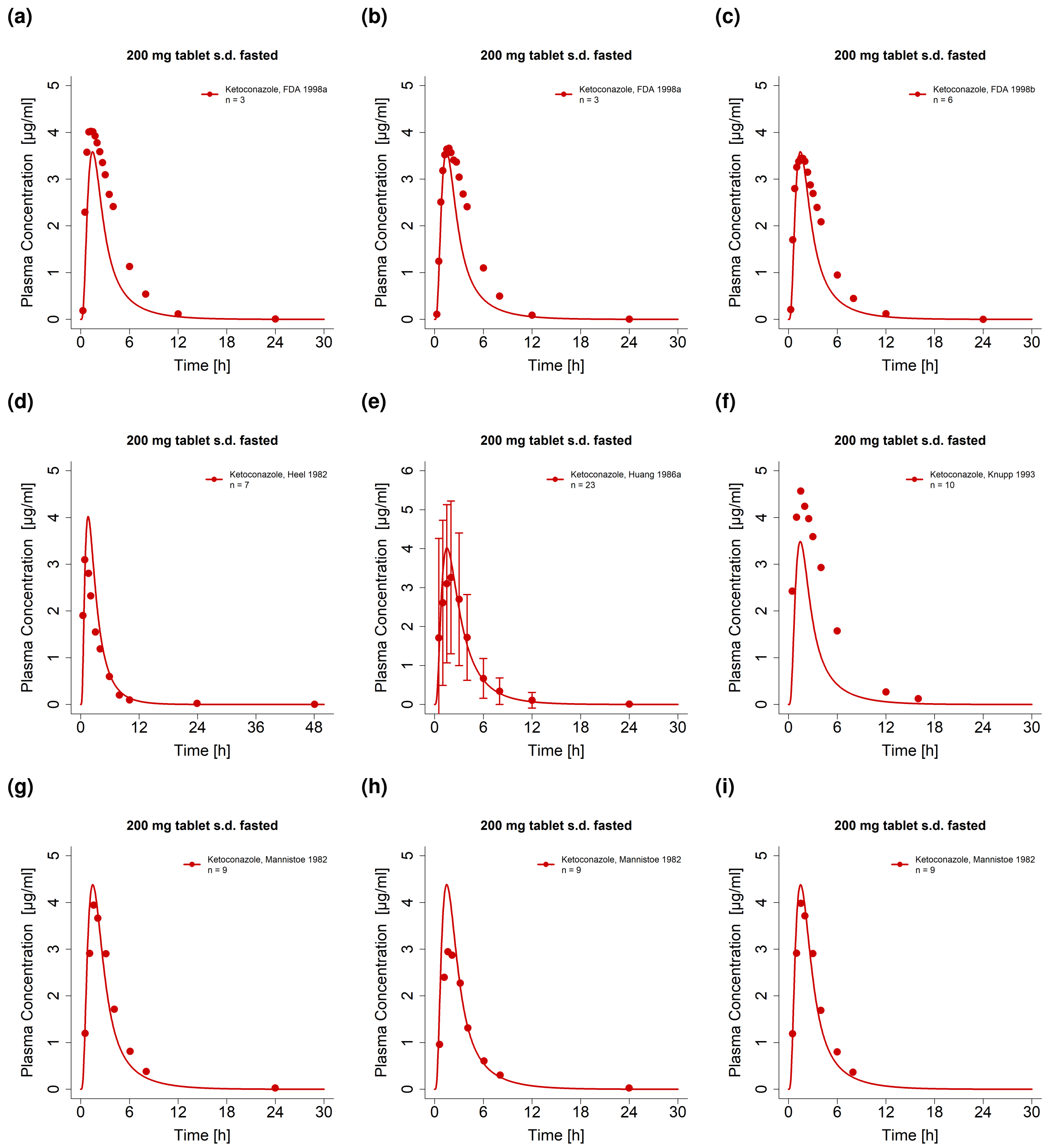

Figure S2.2: Ketoconazole plasma concentration-time profiles. Model predictions are shown as lines, observed data as dots (arithmetic mean ± SD). n: number of individuals studied, s.d: single dose

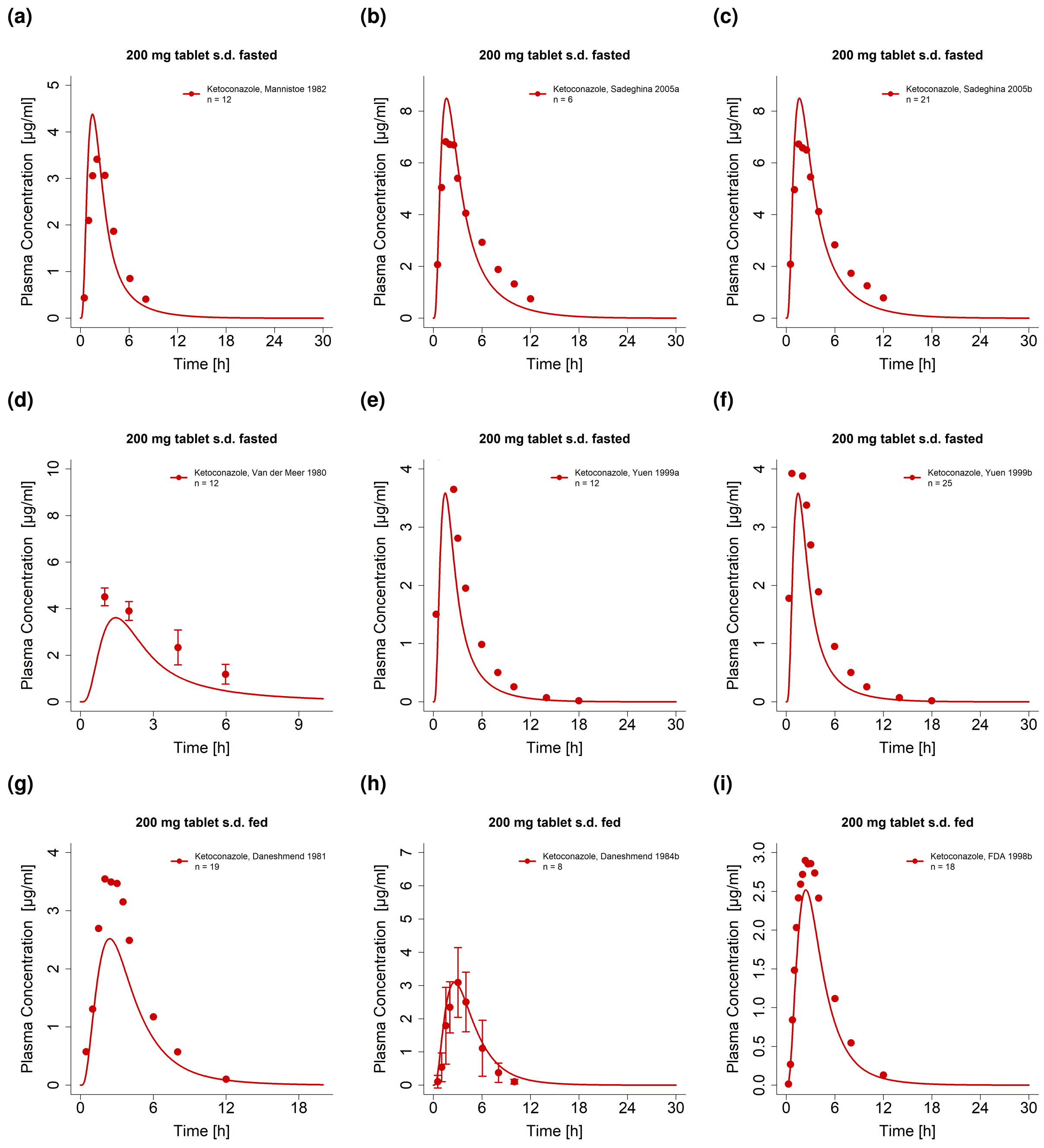

Figure S2.3: Ketoconazole plasma concentration-time profiles. Model predictions are shown as lines, observed data as dots (arithmetic mean ± SD). n: number of individuals studied, s.d: single dose

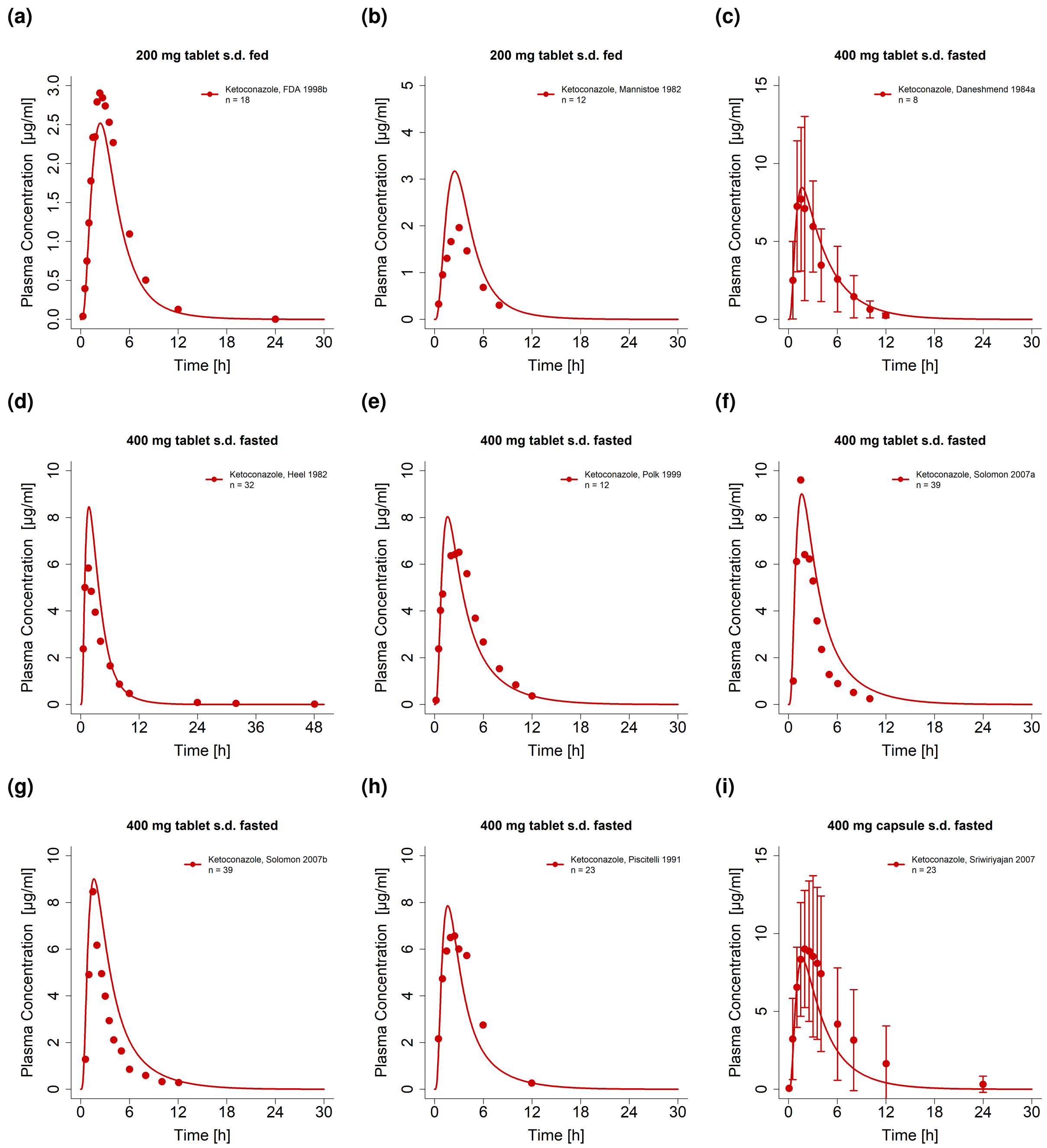

Figure S2.4: Ketoconazole plasma concentration-time profiles. Model predictions are shown as lines, observed data as dots (arithmetic mean ± SD). n: number of individuals studied, s.d: single dose

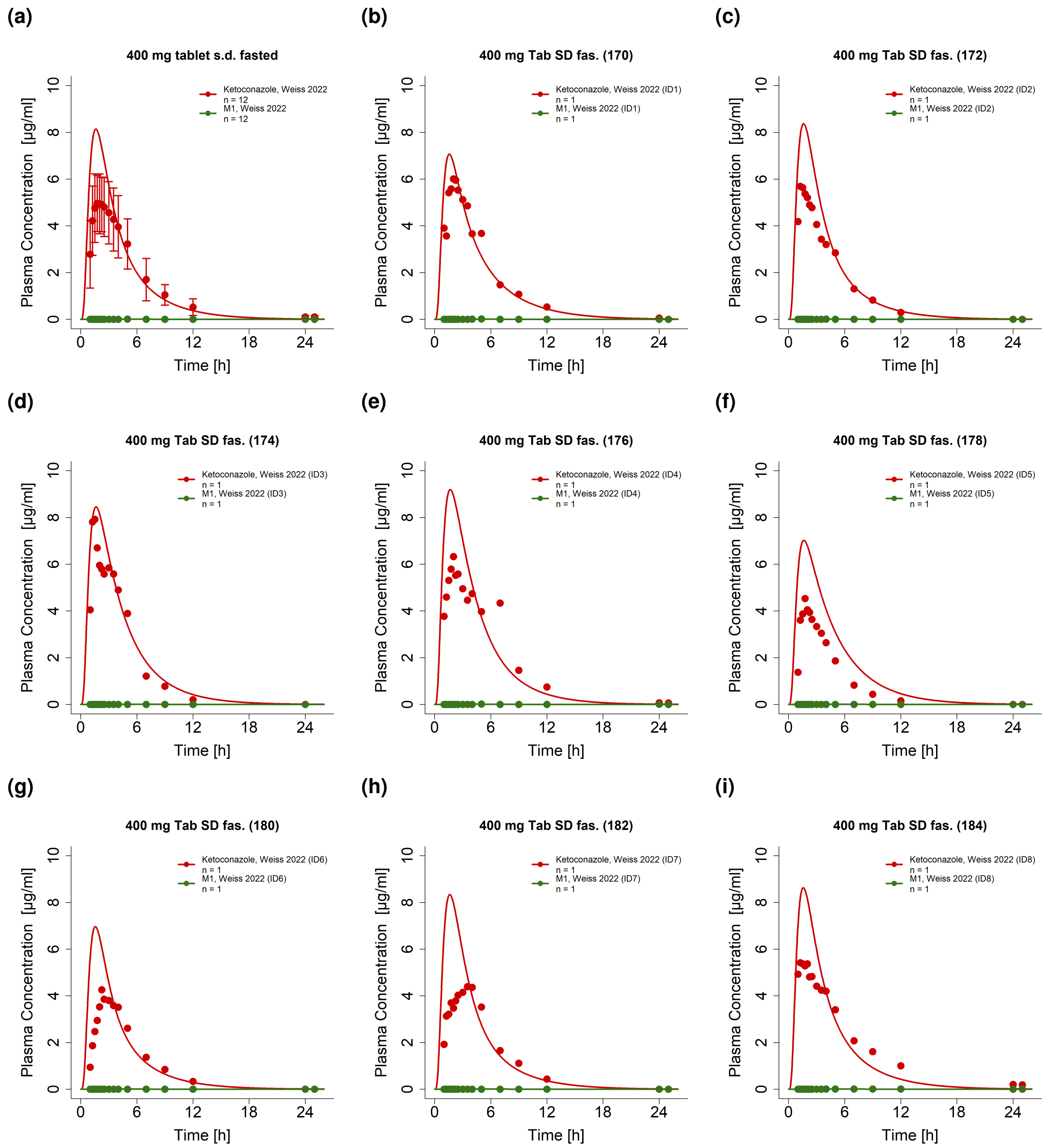

Figure S2.5: Ketoconazole plasma concentration-time profiles. Model predictions are shown as lines, observed data as dots (arithmetic mean ± SD). n: number of individuals studied, s.d: single dose

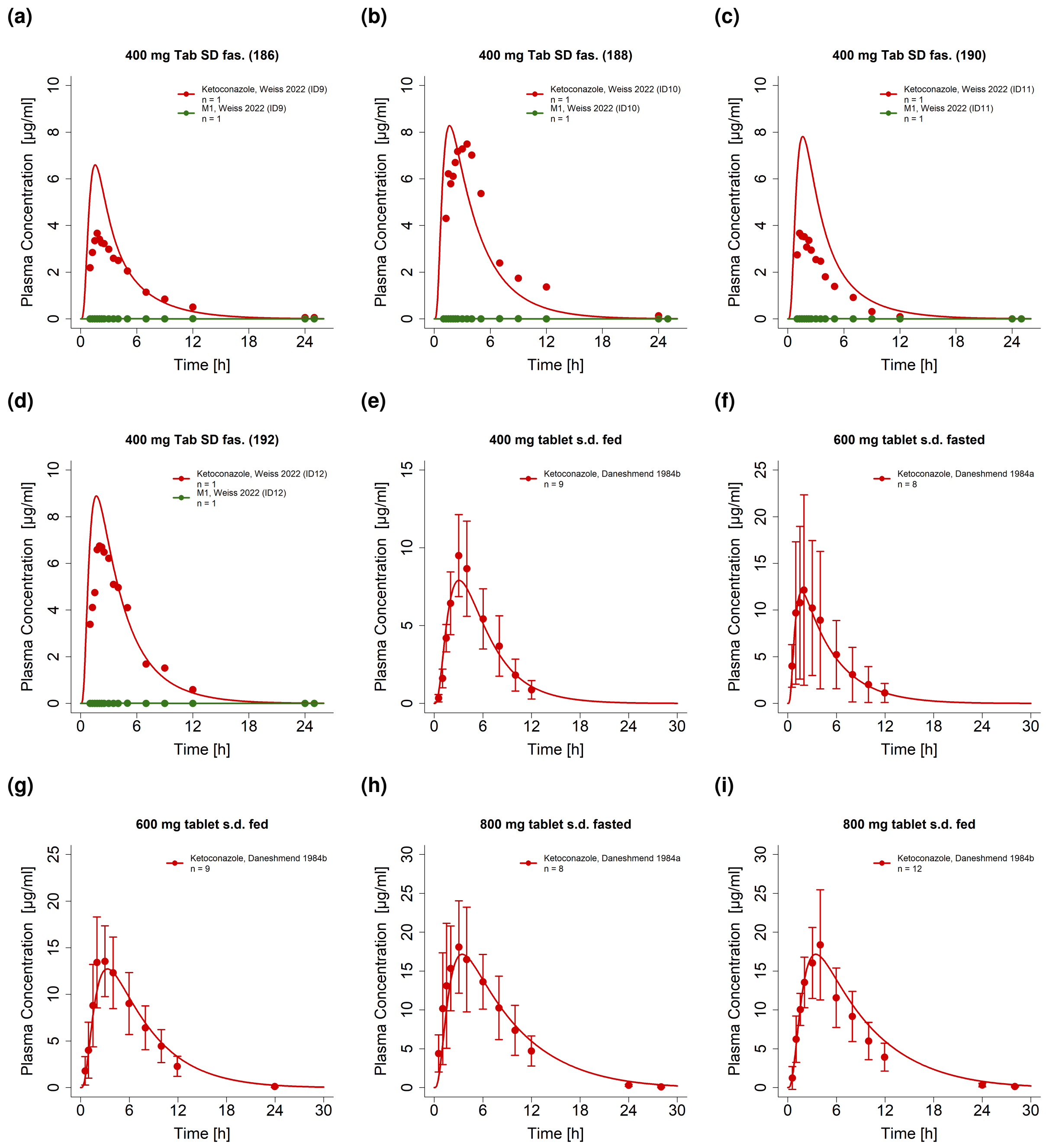

Figure S2.6: Ketoconazole plasma concentration-time profiles. Model predictions are shown as lines, observed data as dots (arithmetic mean ± SD). n: number of individuals studied, s.d: single dose

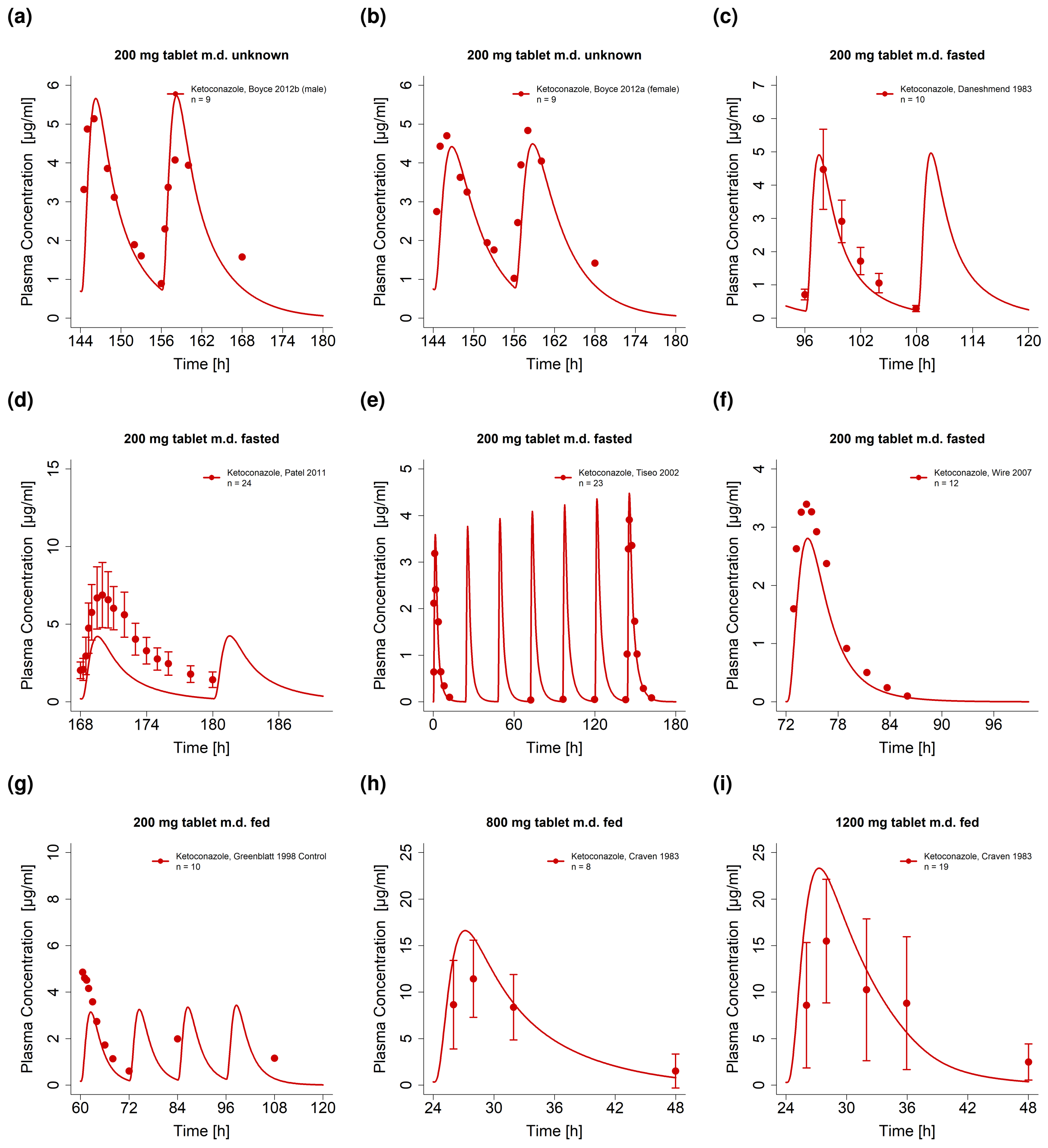

Figure S2.7: Ketoconazole plasma concentration-time profiles. Model predictions are shown as lines, observed data as dots (arithmetic mean ± SD). m.d: multiple dose, n: number of individuals studied, s.d: single dose

S2.2 Plasma concentration-time profiles (Semilogarhitmic)

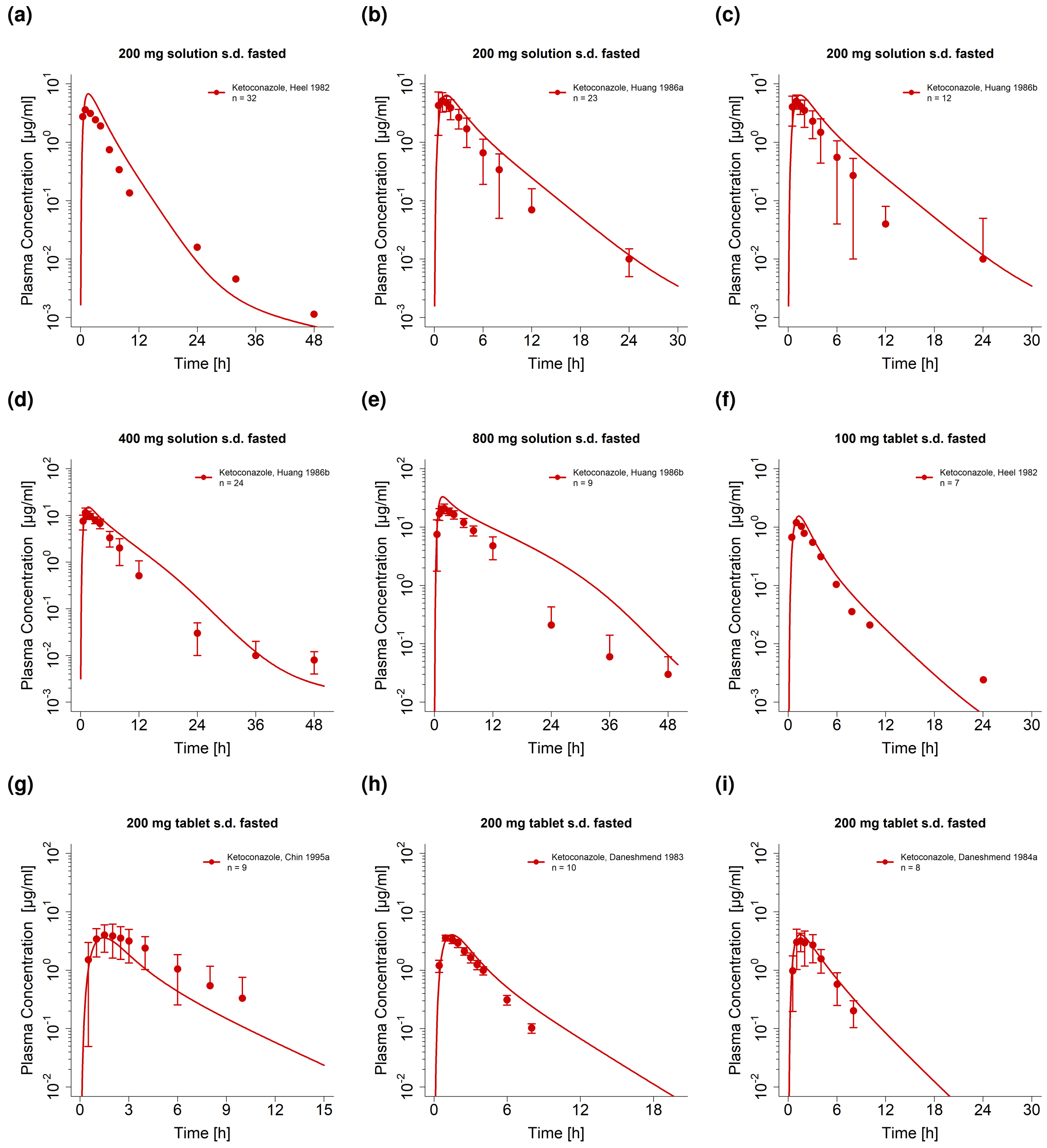

Figure S2.8: Ketoconazole plasma concentration-time profiles. Model predictions are shown as lines, observed data as dots (arithmetic mean ± SD). n: number of individuals studied, s.d: single dose

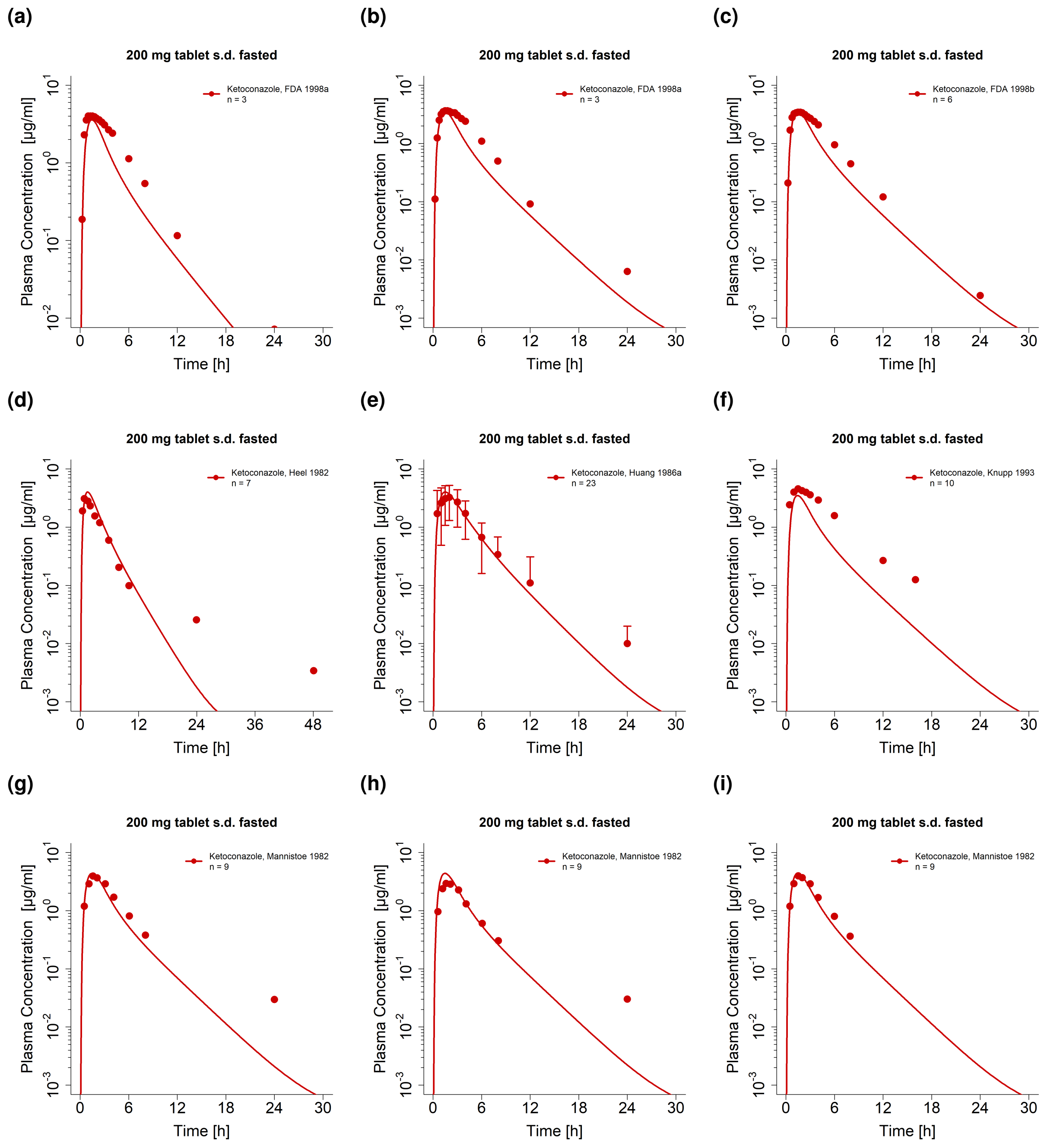

Figure S2.9: Ketoconazole plasma concentration-time profiles. Model predictions are shown as lines, observed data as dots (arithmetic mean ± SD). n: number of individuals studied, s.d: single dose

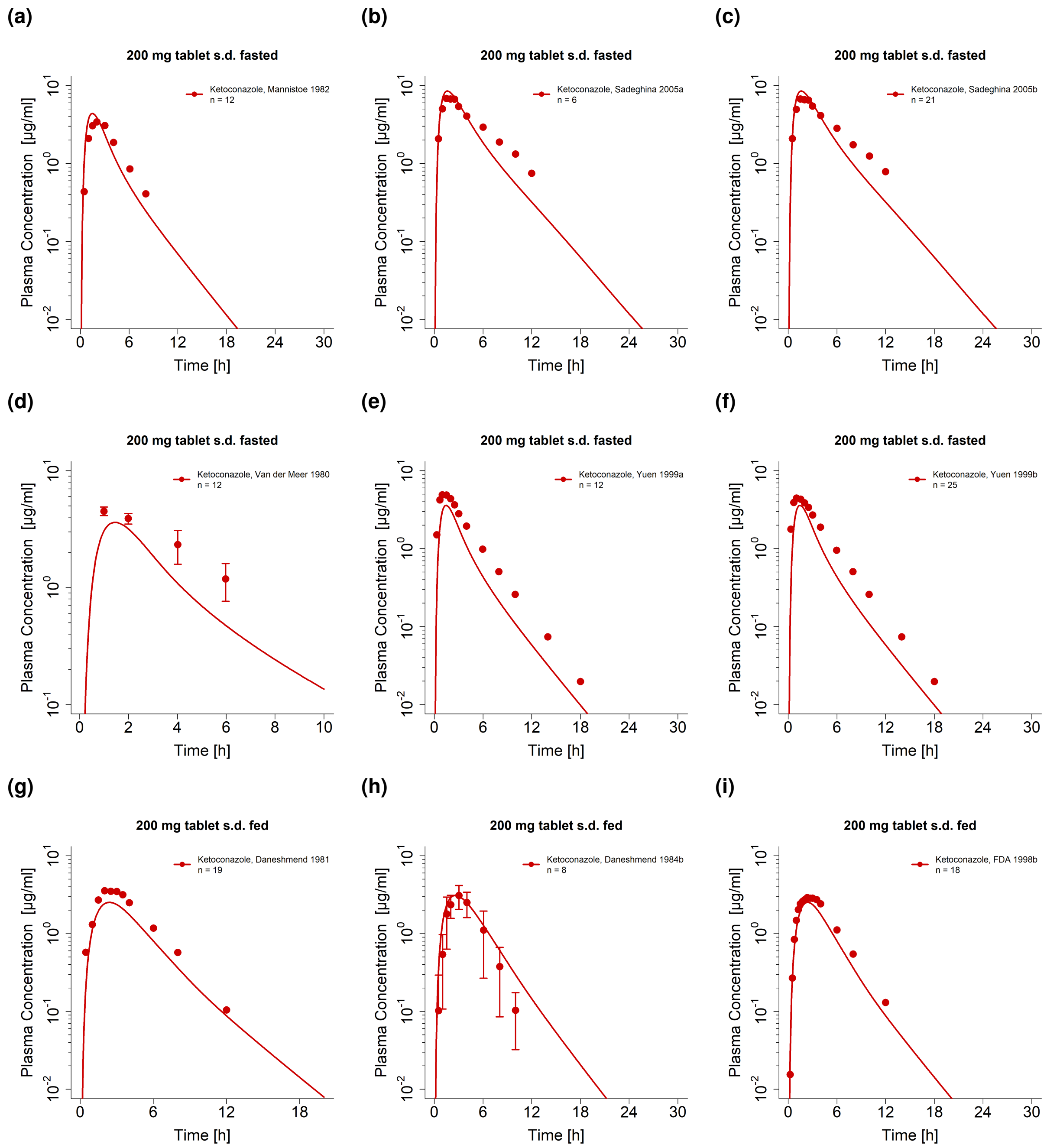

Figure S2.10: Ketoconazole plasma concentration-time profiles. Model predictions are shown as lines, observed data as dots (arithmetic mean ± SD). n: number of individuals studied, s.d: single dose

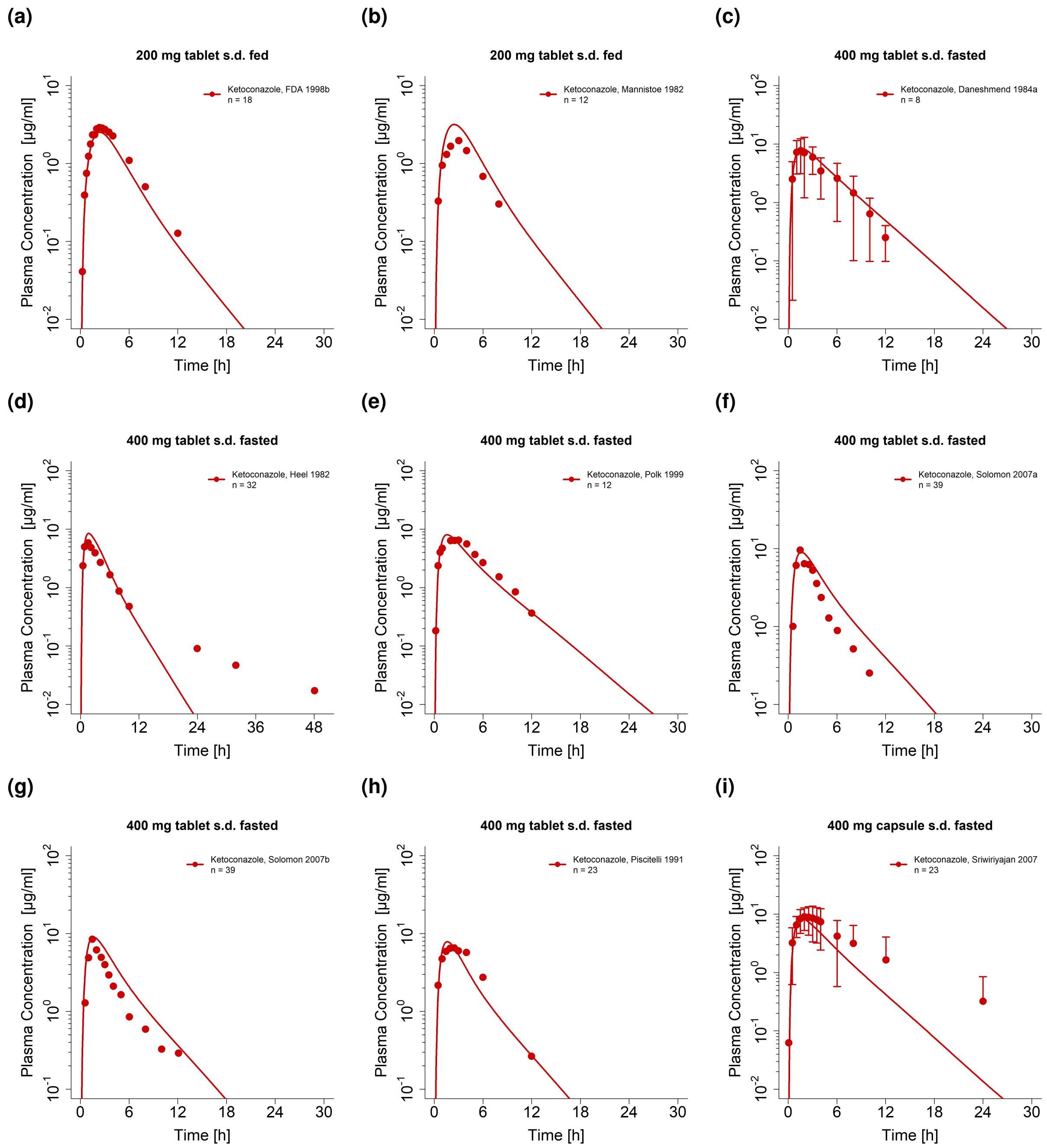

Figure S2.11: Ketoconazole plasma concentration-time profiles. Model predictions are shown as lines, observed data as dots (arithmetic mean ± SD). n: number of individuals studied, s.d: single dose

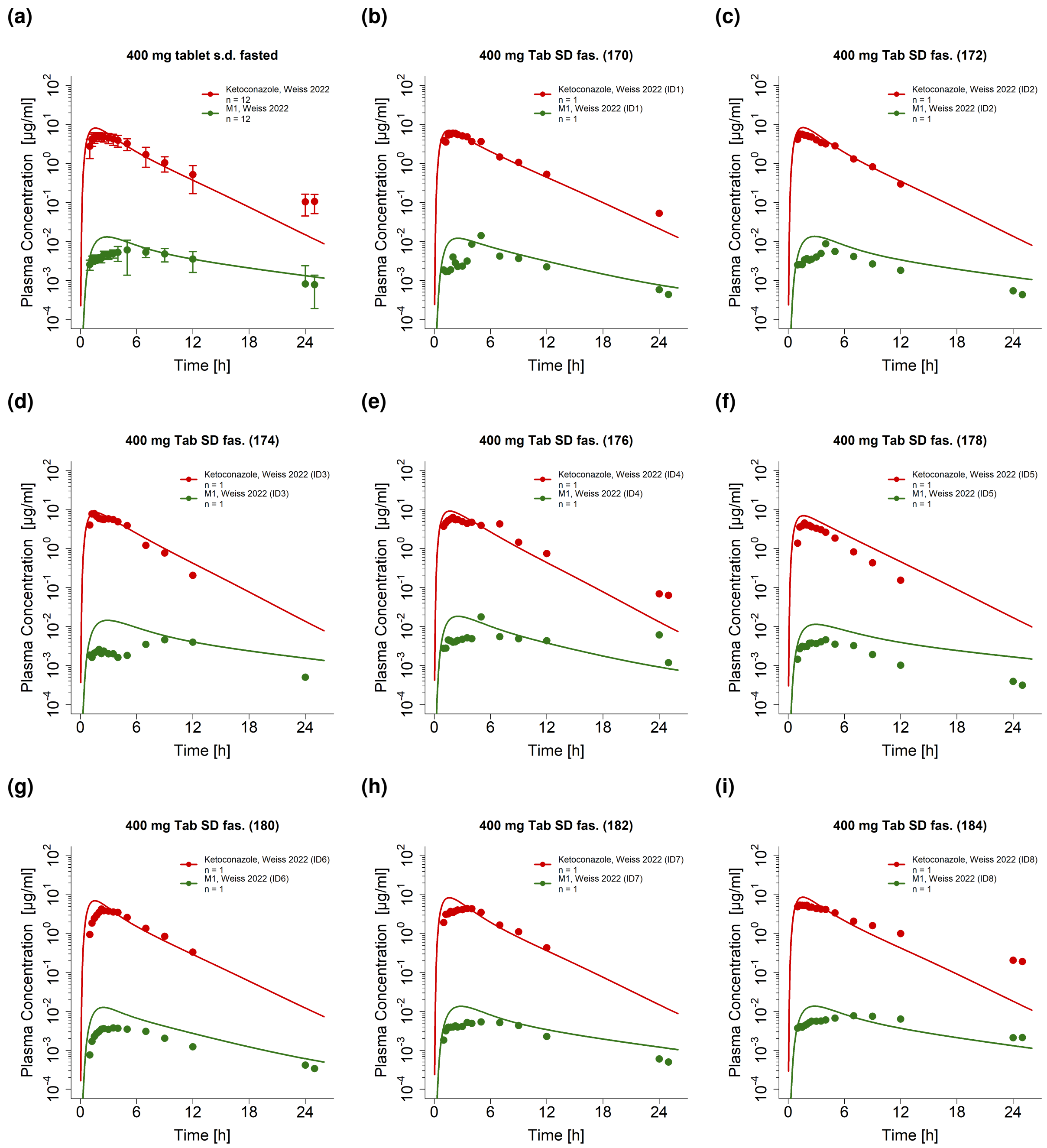

Figure S2.12: Ketoconazole plasma concentration-time profiles. Model predictions are shown as lines, observed data as dots (arithmetic mean ± SD). n: number of individuals studied, s.d: single dose

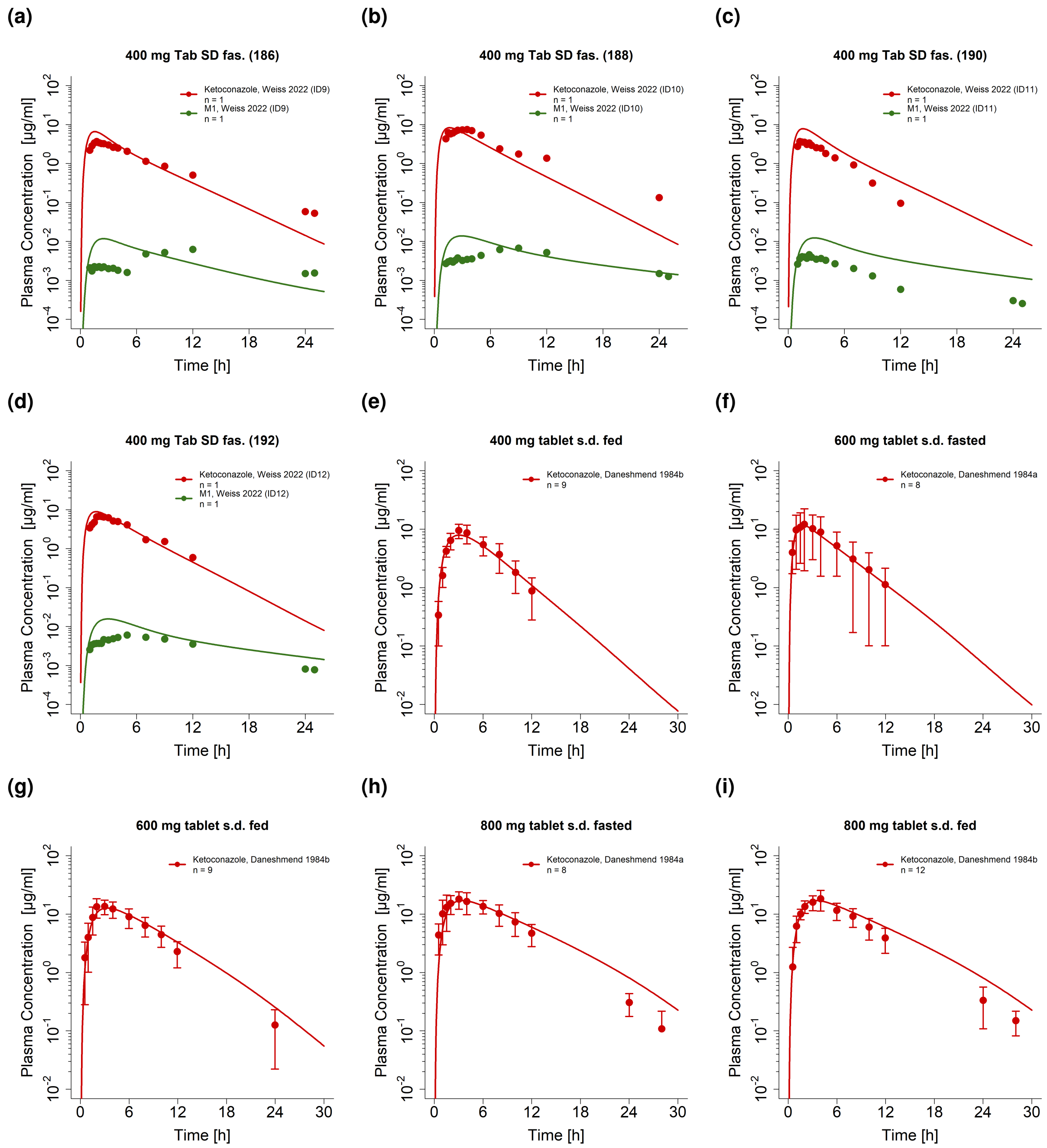

Figure S2.13: Ketoconazole plasma concentration-time profiles. Model predictions are shown as lines, observed data as dots (arithmetic mean ± SD). n: number of individuals studied, s.d: single dose

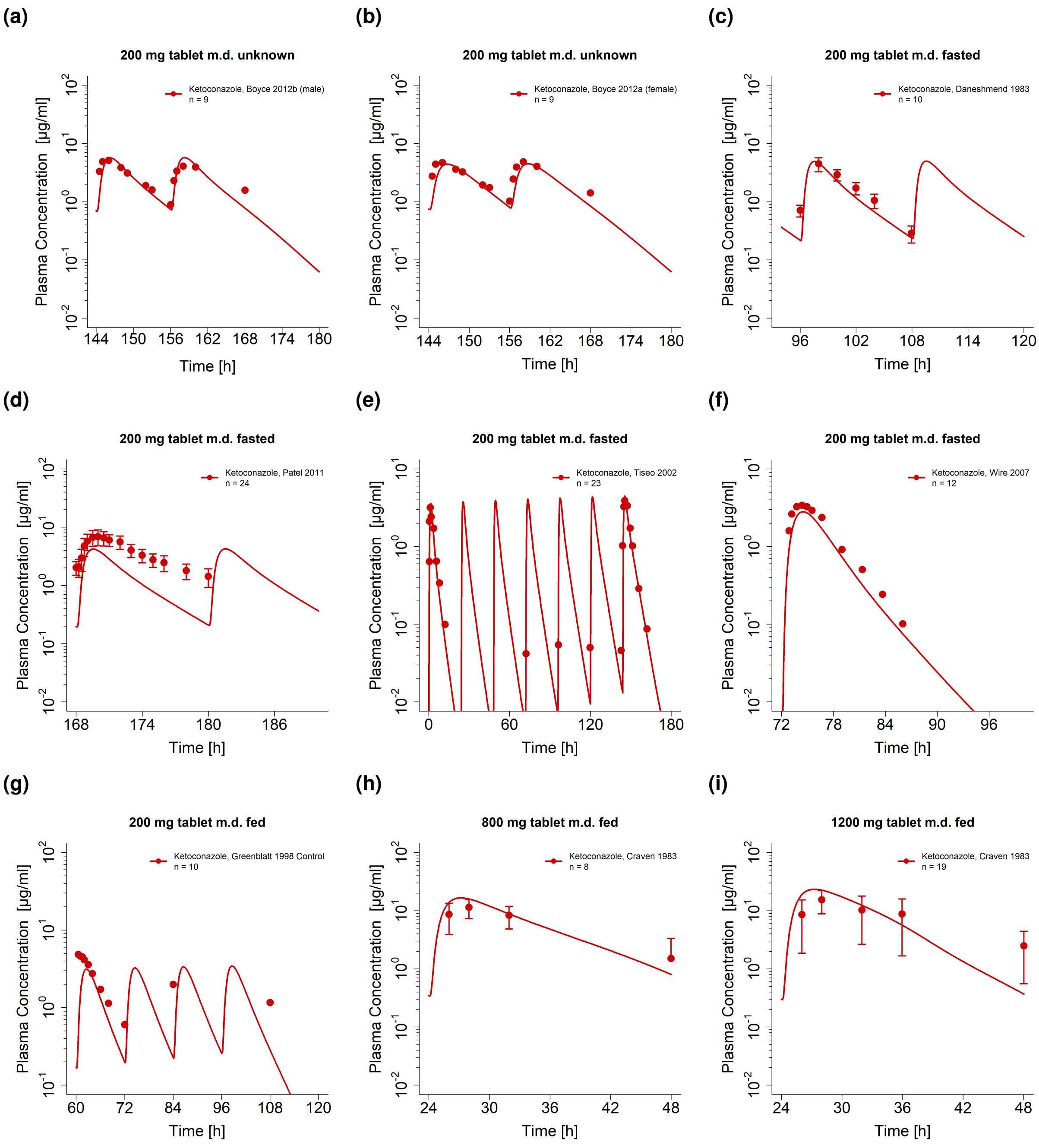

Figure S2.14: Ketoconazole plasma concentration-time profiles. Model predictions are shown as lines, observed data as dots (arithmetic mean ± SD). m.d.: multiple dose, n: number of individuals studied, s.d: single dose

S2.3 Predicted compared to observed concentrations goodness-of-fit plots

(a) Fasted conditions **(b) Fed conditions** **(c) Unknown food intake**

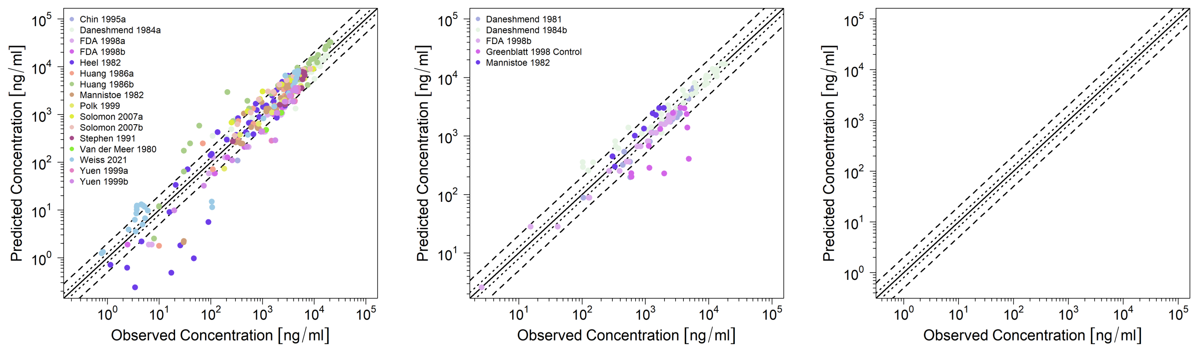

Figure S2.15: Predicted compared to observed ketoconazole plasma concentration values. The solid line marks the line of identity. Dotted lines indicate 1.25-fold, dashed lines indicate 2-fold deviation.

S2.4 AUC_{last} and C_{max} goodness-of-fit plots

(a) AUC_{last} **(b) C_{max}**

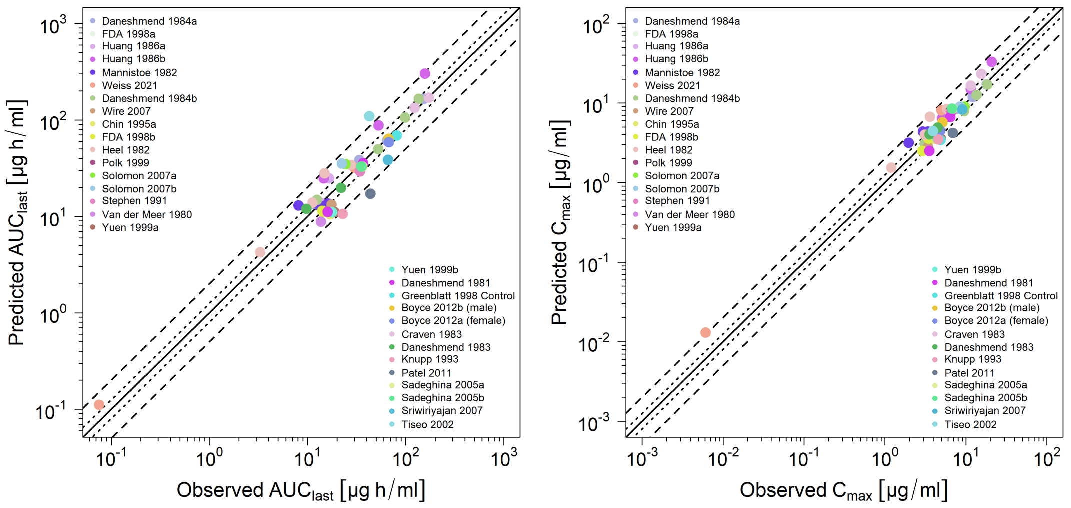

Figure S2.16: Predicted compared to observed ketoconazole AUC_{last} and C_{max} values. The solid line marks the line of identity. Dotted lines indicate 1.25-fold, dashed lines indicate 2-fold deviation. AUC_{last}: area under the plasma concentration-time curve from the time of drug administration to the last concentration measurement, C_{max}: maximum plasma concentration

S2.5 Mean relative deviation of plasma concentration predictions

Table S2.4: Mean relative deviation values of ketoconazole and N-deacetylketoconazole

Dose [mg]	Route	N	DFI	MRD	Dataset	Reference
Ketoconazole						
200	sol s.d	12	fasted	1.71	training	Heel 1982 [12]
200	sol s.d	23	fasted	1.30	test	Huang 1986a [13]
200	sol s.d	12	fasted	1.36	test	Huang 1986b [13]
400	sol s.d	12	fasted	1.63	test	Huang 1986b [13]
800	sol s.d	12	fasted	1.39	training	Huang 1986b [13]
100	tab s.d	12	fasted	2.08	training	Heel 1982 [12]
200	tab s.d	9	fasted	1.09	test	Chin 1995 [14]
200	tab s.d	8	unknown	1.24	test	Daneshmend 1983 [15]
200	tab s.d	8	fasted	1.23	test	Daneshmend 1984a [16]
200	tab s.d	39	fasted	1.48	training	FDA 1998a [17]
200	tab s.d	39	fasted	1.48	test	FDA 1998a [17]
200	tab s.d	23	fasted	1.16	test	FDA 1998b [17]
200	tab s.d	12	fasted	4.39	test	Heel 1982 [12]
200	tab s.d	23	fasted	1.30	test	Huang 1986a [13]
200	tab s.d	12	unknown	1.21	test	Knupp 1993 [18]
200	tab s.d	10	fasted	1.73	training	Mannistoe 1982 [19]
200	tab s.d	10	fasted	1.76	test	Mannistoe 1982 [19]
200	tab s.d	10	fasted	1.10	test	Mannistoe 1982 [19]
200	tab s.d	10	fasted	1.39	test	Mannistoe 1982 [19]
200	tab s.d	12	unknown	1.07	test	Sadeghina 2005 [20]
200	tab s.d	12	unknown	1.06	test	Sadeghina 2005 [20]
200	tab s.d	3	fasted	1.12	test	Van der Meer 1980 [22]
200	tab s.d	18	fasted	1.22	test	Yuen 1999a [23]
200	tab s.d	18	fasted	1.20	test	Yuen 1999b [23]
200	tab s.d	6	fed	1.10	test	Daneshmend 1981 [49]
200	tab s.d	8	fed	1.32	test	Daneshmend 1984b [16]
200	tab s.d	23	fed	1.11	training	FDA 1998b [17]
200	tab s.d	23	fed	1.18	test	FDA 1998b [17]
200	tab s.d	10	fed	1.35	test	Mannistoe 1982 [19]
400	tab s.d	8	fasted	1.18	test	Daneshmend 1984a [16]
400	tab s.d	12	fasted	4.66	test	Heel 1982 [12]
400	tab s.d	12	fasted	1.09	test	Polk 1999 [24]
400	tab s.d	24	fasted	1.38	test	Solomon 2007a [21]
400	tab s.d	24	fasted	1.34	test	Solomon 2007b [21]
400	tab s.d	6	fasted	1.11	test	Piscitelli 1991 [25]
400	cap s.d	12	unknown	3.23	test	Sriwiriyajan 2007 [26]
400	tab s.d	12	fasted	1.16	training	Weiss 2022 [27]
400	tab s.d	8	fed	1.20	test	Daneshmend 1984b [16]
600	tab s.d	8	fasted	1.08	training	Daneshmend 1984a [16]
600	tab s.d	8	fed	1.13	test	Daneshmend 1984b [16]
800	tab s.d	8	fasted	1.24	test	Daneshmend 1984a [16]
800	tab s.d	8	fed	1.18	training	Daneshmend 1984b [16]
200	tab m.d	24	unknown	1.16	training	Boyce 2012b [28]
200	tab m.d	24	unknown	1.12	test	Boyce 2012a [28]
200	tab m.d	8	unknown	1.07	test	Daneshmend 1983 [15]
200	tab m.d	15	unknown	1.39	test	Patel 2011 [29]
200	tab s.d	21	unknown	1.24	test	Tiseo 2002 [30]
200	tab m.d	15	unknown	1.09	training	Wire 2007 [31]
400	tab m.d	9	fed	1.39	test	Greenblatt 1998 Control [32]
800	tab m.d	2	unknown	1.05	test	Craven 1983 [33]

(Continued on next page...)

Table S2.4: Mean relative deviation values of ketoconazole and N-deacetylketoconazole *(continued)*

Dose [mg]	Route	N	DFI	MRD	Dataset	Reference
1200	tab m.d	2	unknown	1.23	test	Craven 1983 [33]
N-Deacetylketoconazole						
400	tab s.d	12	fasted	2.51	training	Weiss 2021 [27]
**Mean MRD for ketoconazole**				**1.42**		
**Mean MRD for N-deacetylketoconazole**				**2.51**		
**Overall mean MRD (range)**				**1.45 (1.09–2.69)**		
				**92.45% (49/53) $\leq$ 2**		

cap: capsule, DFI: drug-food-interaction, m.d: multiple dose, MRD: mean relative deviation, N: number of individuals studied, Route: route of administration, s.d: single dose, sol: solution, tab: tablet

S2.6 Geometric mean fold error of predicted AUC_{last} and C_{max} values

Table S2.5: Predicted and observed AUC_{last} and C_{max} values of ketoconazole and N-deacetylketoconazole

Dose [mg]	Route	N	DFI	AUC_{last} [µg*h/ml]			C_{max} [µg/ml]			Dataset	Reference
				Pred	Obs	Pred/Obs	Pred	Obs	Pred/Obs		
Ketoconazole											
200	sol s.d	12	fasted	28.07	14.89	1.88	6.74	3.59	1.88	training	Heel 1982 [12]
200	sol s.d	23	fasted	24.91	16.58	1.50	6.40	5.16	1.24	test	Huang 1986a [13]
200	sol s.d	12	fasted	24.91	14.60	1.71	6.40	5.04	1.27	test	Huang 1986b [13]
400	sol s.d	12	fasted	87.95	52.96	1.66	15.09	11.46	1.32	test	Huang 1986b [13]
800	sol s.d	12	fasted	303.31	156.86	1.93	33.11	20.88	1.59	training	Huang 1986b [13]
100	tab s.d	12	fasted	4.27	3.29	1.30	1.54	1.20	1.29	training	Heel 1982 [12]
200	tab s.d	9	fasted	10.59	16.92	0.63	3.59	4.00	0.90	test	Chin 1995 [14]
200	tab s.d	8	unknown	11.99	9.73	1.23	4.04	3.56	1.13	test	Daneshmend 1983 [15]
200	tab s.d	8	fasted	13.45	11.63	1.16	4.07	3.18	1.28	test	Daneshmend 1984a [16]
200	tab s.d	39	fasted	11.12	18.68	0.60	3.59	4.02	0.89	training	FDA 1998a [17]
200	tab s.d	39	fasted	11.12	17.16	0.65	3.58	3.66	0.98	test	FDA 1998a [17]
200	tab s.d	23	fasted	11.12	15.94	0.70	3.58	3.44	1.04	test	FDA 1998b [17]
200	tab s.d	12	fasted	13.87	11.25	1.23	4.02	3.10	1.30	test	Heel 1982 [12]
200	tab s.d	23	fasted	13.79	13.76	1.00	4.02	3.26	1.23	test	Huang 1986a [13]
200	tab s.d	12	unknown	10.66	22.84	0.47	3.49	4.56	0.76	test	Knupp 1993 [18]
200	tab s.d	10	fasted	13.63	16.14	0.84	4.38	3.95	1.11	training	Mannistoe 1982 [19]
200	tab s.d	10	fasted	13.61	12.46	1.09	4.38	2.94	1.49	test	Mannistoe 1982 [19]
200	tab s.d	10	fasted	12.84	13.65	0.94	4.38	3.98	1.10	test	Mannistoe 1982 [19]
200	tab s.d	10	fasted	12.89	13.24	0.97	4.38	3.41	1.28	test	Mannistoe 1982 [19]
200	tab s.d	12	unknown	32.84	36.04	0.91	8.50	6.82	1.25	test	Sadeghina 2005 [20]
200	tab s.d	12	unknown	32.84	35.31	0.93	8.50	6.73	1.26	test	Sadeghina 2005 [20]
200	tab s.d	3	fasted	8.82	13.69	0.64	3.61	4.51	0.80	test	Van der Meer 1980 [22]
200	tab s.d	18	fasted	11.08	18.89	0.59	3.58	4.91	0.73	test	Yuen 1999a [23]
200	tab s.d	18	fasted	11.08	17.76	0.62	3.58	4.46	0.80	test	Yuen 1999b [23]
200	tab s.d	6	fed	11.11	15.96	0.70	2.52	3.55	0.71	test	Daneshmend 1981 [49]
200	tab s.d	8	fed	14.88	12.50	1.19	3.09	3.09	1.00	test	Daneshmend 1984b [16]
200	tab s.d	23	fed	11.13	14.40	0.77	2.52	2.90	0.87	training	FDA 1998b [17]
200	tab s.d	23	fed	11.41	14.14	0.81	2.52	2.90	0.87	test	FDA 1998b [17]
200	tab s.d	10	fed	12.99	8.10	1.60	3.17	1.96	1.61	test	Mannistoe 1982 [19]
400	tab s.d	8	fasted	38.44	33.67	1.14	8.46	7.71	1.10	test	Daneshmend 1984a [16]
400	tab s.d	12	fasted	34.20	27.62	1.24	8.46	5.83	1.45	test	Heel 1982 [12]
400	tab s.d	12	fasted	32.79	35.69	0.92	8.04	6.52	1.23	test	Polk 1999 [24]
200	tab s.d	24	fasted	34.90	24.30	1.44	9.01	9.61	0.94	test	Solomon 2007a [21]

(Continued on next page...)

Table S2.5: Mean relative deviation values of ketoconazole and N-deacetylketoconazole *(continued)*

Dose [mg]	Route	N	DFI	AUC_{last} [µg*h/ml]			C_{max} [µg/ml]			Dataset	Reference
				Pred	Obs	Pred/Obs	Pred	Obs	Pred/Obs		
200	tab s.d	24	fasted	35.46	22.40	1.58	9.01	8.46	1.06	test	Solomon 2007b [21]
400	tab s.d	6	fasted	29.48	34.32	0.86	7.86	6.57	1.20	test	Piscitelli 1991 [25]
400	cap s.d	12	unknown	38.61	65.85	0.59	8.34	9.02	0.93	test	Sriwiriyajan 2007 [26]
400	tab s.d	12	fasted	32.05	30.02	1.07	8.14	4.96	1.64	training	Weiss 2022 [27]
400	tab s.d	8	fed	35.64	36.91	0.97	6.70	6.44	1.04	test	Daneshmend 1984b [16]
600	tab s.d	8	fasted	61.56	64.92	0.95	11.93	12.15	0.98	training	Daneshmend 1984a [16]
600	tab s.d	8	fed	106.05	98.72	1.07	12.73	13.55	0.94	test	Daneshmend 1984b [16]
800	tab s.d	8	fasted	166.39	153.04	1.09	17.15	18.09	0.95	test	Daneshmend 1984a [16]
800	tab s.d	8	fed	166.38	135.94	1.22	17.15	18.37	0.93	training	Daneshmend 1984b [16]
200	tab m.d	24	unknown	58.75	67.41	0.87	4.49	4.83	0.93	training	Boyce 2012b [28]
200	tab m.d	24	unknown	63.94	66.77	0.96	5.73	5.14	1.12	test	Boyce 2012a [28]
200	tab m.d	8	unknown	19.74	21.94	0.90	4.91	4.47	1.10	test	Daneshmend 1983 [15]
200	tab m.d	15	unknown	17.19	43.67	0.39	4.21	6.87	0.61	test	Patel 2011 [29]
200	tab s.d	21	unknown	109.29	42.47	2.57	4.48	3.91	1.15	test	Tiseo 2002 [30]
200	tab m.d	15	unknown	13.25	17.51	0.76	2.81	3.40	0.83	training	Wire 2007 [31]
400	tab m.d	9	fed	68.89	81.00	0.85	3.44	4.86	0.71	test	Greenblatt 1998 Control [32]
800	tab m.d	2	unknown	170.53	172.40	0.99	23.32	15.48	1.51	test	Craven 1983 [33]
1200	tab m.d	2	unknown	134.16	123.24	1.09	16.61	11.43	1.45	test	Craven 1983 [33]
N-Deacetylketoconazole											
400	tab s.d	12	fasted	0.11	0.08	1.48	0.01	0.01	2.15	training	Weiss 2021 [27]
**Mean GMFE for ketoconazole**						**1.37**			**1.24**		
**Mean GMFE for N-deacetylketoconazole**						**1.48**			**2.15**		
**Overall mean GMFE (range)**						**1.37 (1.00–2.57)**			**1.26 (1.00–2.15)**		
						**94.34% (50/53) $\leq$ 2**			**98.11% (52/53) $\leq$ 2**		

AUC_{last}: area under the plasma concentration-time curve calculated from the first to last time point of measurement, cap: capsule, C_{max}: maximum plasma concentration, DFI: drug-food-interaction, GMFE: geometric mean fold error, m.d: multiple dose, N: number of individuals studied, obs: observed, pred: predicted, Route: route of administration, s.d: single dose, sol: solution, tab: tablet

S2.7 Ketoconazole – DFI model evaluation

S2.7.1 Plasma concentration-time profiles (Linear)

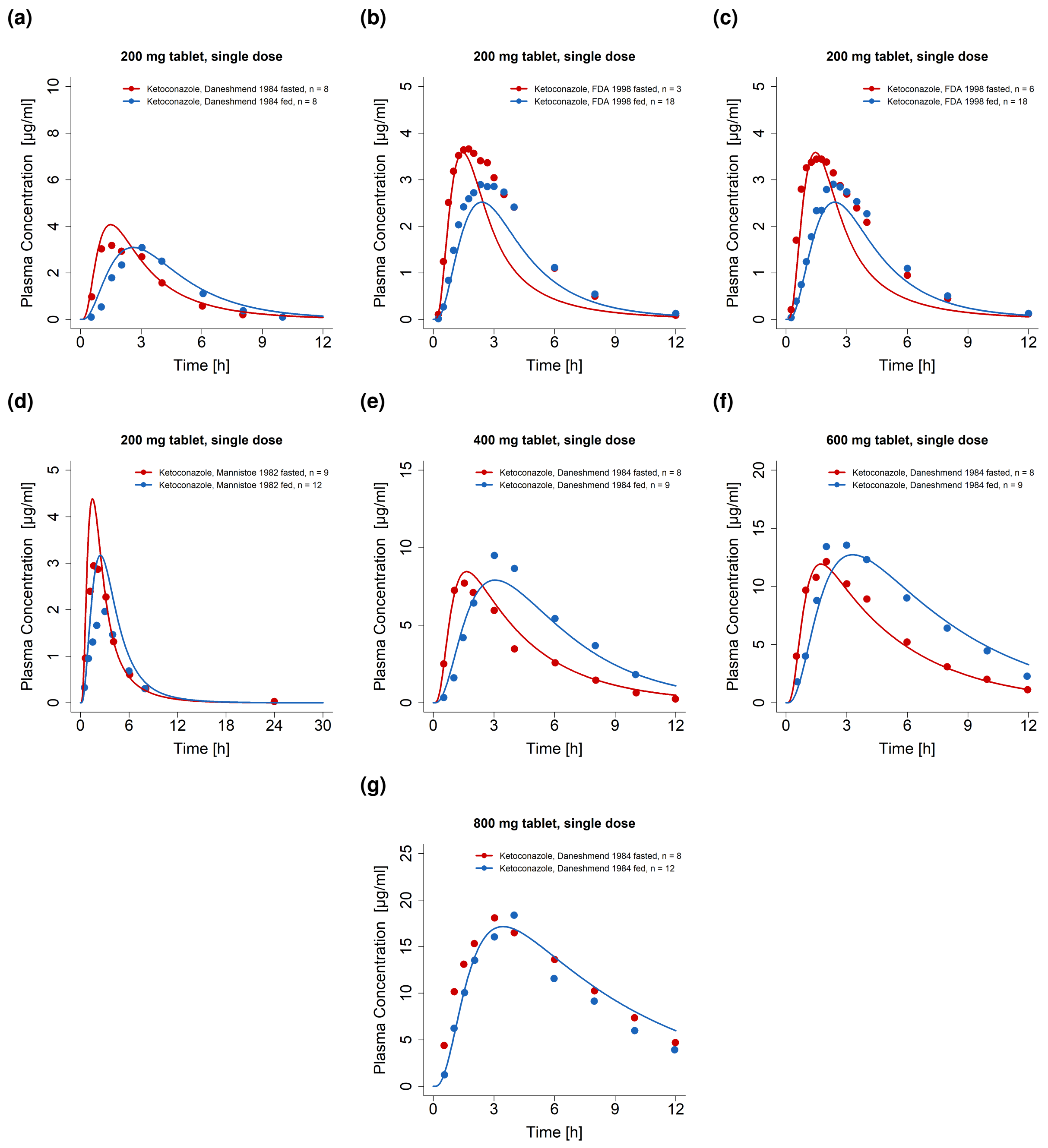

Figure S2.17: Comparison of predicted and observed ketoconazole plasma concentration-time profiles under fasted and fed conditions. Model predictions are shown as lines, observed data as dots (arithmetic mean ± SD). n: number of individuals studied.

S2.7.2 Plasma concentration-time profiles (Semilogarhitmic)

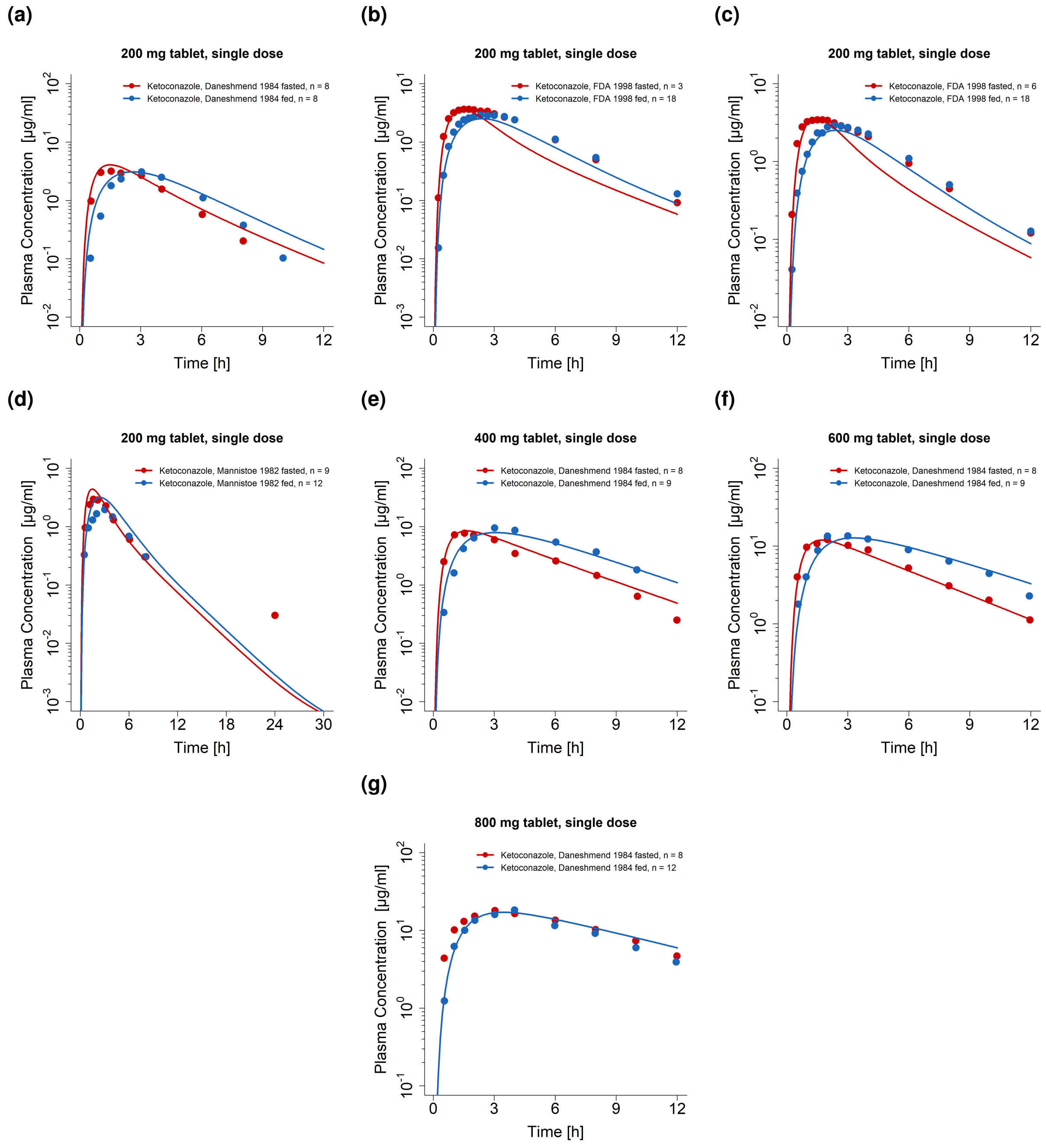

Figure S2.18: Comparison of predicted and observed ketoconazole plasma concentration-time profiles under fasted and fed conditions. Model predictions are shown as lines, observed data as dots (arithmetic mean ± SD). n: number of individuals studied.

S2.7.3 DFI AUC_{last} and DFI C_{max} goodness-of-fit plots

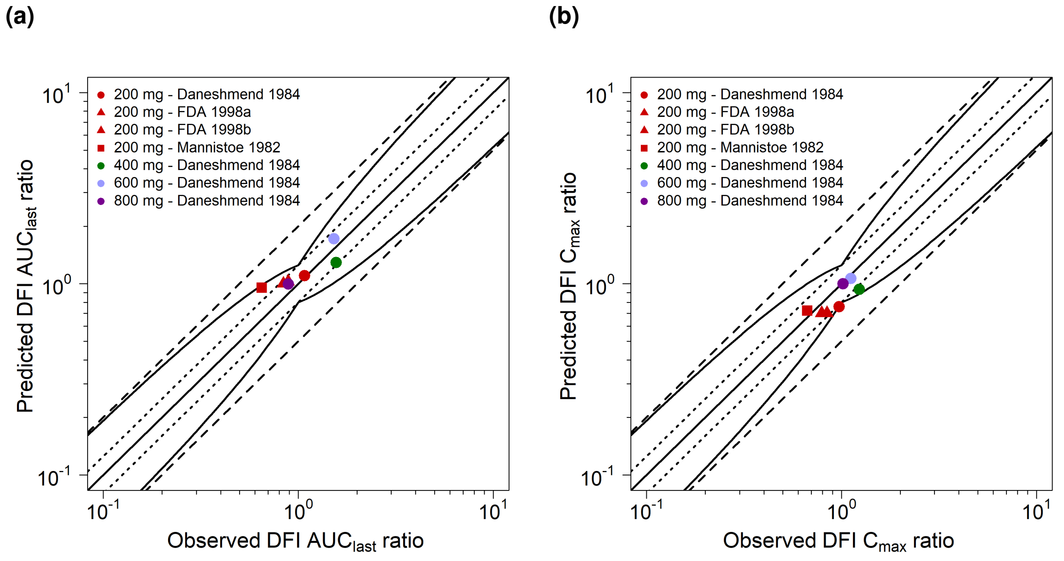

Figure S2.19: Predicted compared to observed DFI AUC_{last} and C_{max} ratios. The straight solid line marks the line of identity. The curved solid lines show the prediction acceptance limits proposed by Guest et al. including 1.25-fold variability [50]. Dotted lines indicate 1.25-fold, dashed lines indicate 2-fold deviation. AUC_{last}: area under the plasma concentration-time curve from the time of drug administration to the last concentration measurement, C_{max}: maximum plasma concentration, DFI: drug-food interaction

S2.7.4 Geometric mean fold error of predicted AUC_{last} and C_{max} values

Table S2.6: Predicted and observed DFI AUC_{last} and C_{max} ratios of ketoconazole

Dose [mg]	Route	N	DFI AUC_{last} ratio			DFI C_{max} ratio			Reference
			Pred	Obs	Pred/Obs	Pred	Obs	Pred/Obs	
200	tab s.d	8	1.11	1.08	1.03	0.76	0.97	0.78	Daneshmend 1984 [16]
200	tab s.d	39	1.00	0.84	1.19	0.70	0.79	0.89	FDA 1998 a [17]
200	tab s.d	23	1.03	0.89	1.16	0.70	0.84	0.83	FDA 1998 b [17]
200	tab s.d	10	0.95	0.65	1.47	0.72	0.67	1.08	Mannistoe 1982 [19]
400	tab s.d	8	1.29	1.56	0.83	0.94	1.23	0.76	Daneshmend 1984 [16]
600	tab s.d	8	1.72	1.52	1.13	1.07	1.12	0.96	Daneshmend 1984 [16]
800	tab s.d	8	1.00	0.89	1.13	1.00	1.02	0.98	Daneshmend 1984 [16]
**Overall mean GMFE (range)**					**1.19 (1.02–1.47)**			**1.15 (1.02–1.32)**	
					**100.00% (7/7) $\leq$ 2**			**100.00% (7/7) $\leq$ 2**	

AUC_{last}: area under the plasma concentration-time curve calculated from the first to last time point of measurement, cap: capsule, C_{max}: maximum plasma concentration, DFI: drug-food-interaction, GMFE: geometric mean fold error, n: number of individuals studied, obs: observed, pred: predicted, Route: route of administration, tab: tablet, s.d: single dose

S2.8 Sensitivity Analyses

Figures S2.20 and S2.21 show the results of the local sensitivity analyses on the AUC of the compounds ketoconazole and N-deacetylketoconazole. Sensitivity of the model to single parameter changes was determined after the last application of a 7 day multiple dose regimen of 200 mg once daily.

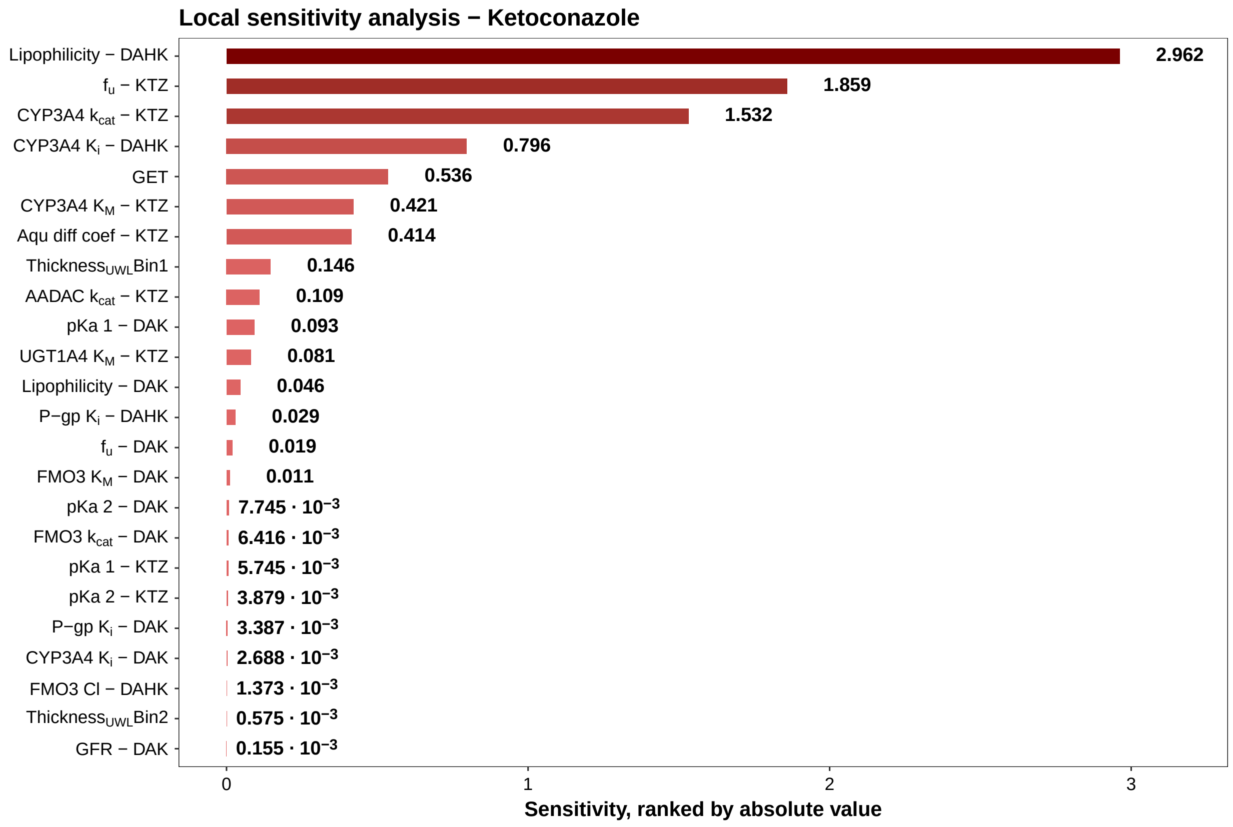

Figure S2.20: Local sensitivity analysis of ketoconazole PBPK model– calculated for sensitivity on ketoconazole plasma AUC_{inf}. AADAC: arylacetamide deacetylase, Aqu. diff. coef.: aqueous diffusion coeffecient, AUC_{inf}: area under the plasma concentration-time curve from the time of the last drug administration extrapolated to infinity, Cl: clearance, CYP: cytochrome P450, DAHK: N-deacetyl-N-hydroxyketoconazole, DAK: N-deacetylketoconazole, FMO: flavin-containing monooxygenase, f_u: fraction unbound, GET: gastric emptying time, GFR: fraction of glomerular filtration rate, k_{cat}: catalytic rate constant, K_i: concentration for half-maximal inhibition, K_M: Michaelis-Menten constant, pKa: acidic dissociation constant, P-gp: P-glycoprotein, $Thickness_{UWL}$: thickness of unstirred water layer for particle radii of the respective bin, UGT: uridine diphosphate glucuronosyltransferase

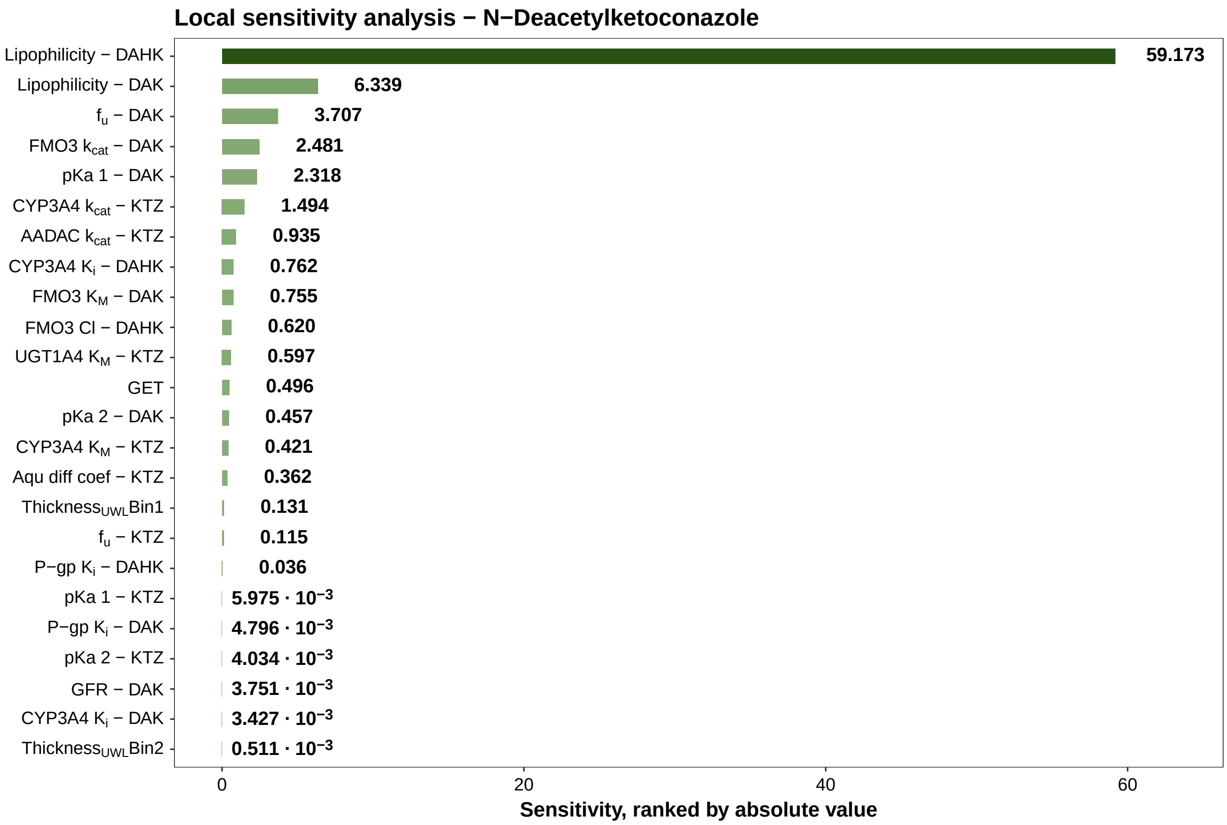

Figure S2.21: Local sensitivity analysis of ketoconazole PBPK model– calculated for sensitivity on N-deacetlyketoconazole plasma AUC_{inf}. AADAC: arylacetamide deacetylase, Aqu. diff. coef.: aqueous diffusion coefficient, AUC_{inf}: area under the plasma concentration-time curve from the time of the last drug administration extrapolated to infinity, Cl: clearance, CYP: cytochrome P450, DAHK: N-deacetyl-N-hydroxyketoconazole, DAK: N-deacetylketoconazole, FMO: flavin-containing monooxygenase, f_u: fraction unbound, GET: gastric emptying time, GFR: fraction of glomerular filtration rate, k_{cat}: catalytic rate constant, K_i: concentration for half-maximal inhibition, K_M: Michaelis-Menten constant, pKa: acidic dissociation constant, P-gp: P-glycoprotein, $Thickness_{UWL}$: thickness of unstirred water layer for particle radii of the respective bin, UGT: uridine diphosphate glucuronosyltransferase

S3 Ketoconazole – DDI Modeling

S3.1 Ketoconazole – Clinical studies

Table S3.7: Clinical study data used for ketoconazole DDI model development

Drug administration		Dose gap [h]	N	Age [years]	Weight [kg]	Females [%]	Dataset	Reference
Ketoconazole	Victim							
	Alfentanil							
-	1 mg iv bol seq (D0)	-	6	28 (21–33)	79 (63–106)	50.00	test	Kharash 2011 Control [51]
400 mg po tab qd (D1–D4)	0.5 mg iv bol seq (D5)	+8	6	28 (21–33)	79 (63–106)	50.00	test	Kharash 2011 DDI [51]
-	1 mg iv bol sim (D0)	-	6	28 (21–33)	79 (63–106)	50.00	test	Kharash 2011 Control [51]
400 mg po tab qd (D1–D4)	0.5 mg iv bol sim (D5)	+8	6	28 (21–33)	79 (63–106)	50.00	training	Kharash 2011 DDI [51]
-	4 mg po tab seq (D0)	-	6	28 (21–33)	79 (63–106)	50.00	test	Kharash 2011 Control [51]
400 mg po tab qd (D1–D4)	1 mg po tab seq (D5)	+8	6	28 (21–33)	79 (63–106)	50.00	test	Kharash 2011 DDI [51]
-	4 mg po tab sim (D0)	-	6	28 (21–33)	79 (63–106)	50.00	test	Kharash 2011 Control [51]
400 mg po tab qd (D1–D4)	1 mg po tab sim (D5)	+8	6	28 (21–33)	79 (63–106)	50.00	test	Kharash 2011 DDI [51]
-	1 mg iv bol & 4 mg po tab s.d. seq (D0)	-	6	28 (21–33)	79 (63–106)	50.00	test	Kharash 2011 Control [51]
400 mg po tab qd (D1–D4)	0.5 mg iv bol & 1 mg po tab seq (D5)	+8	6	28 (21–33)	79 (63–106)	50.00	test	Kharash 2011 DDI [51]
-	1 mg iv bol & 4 mg po tab s.d. sim (D0)	-	6	28 (21–33)	79 (63–106)	50.00	test	Kharash 2011 Control [51]
400 mg po tab qd (D1–D4)	0.5 mg iv bol & 1 mg po tab sim (D5)	+8	6	28 (21–33)	79 (63–106)	50.00	test	Kharash 2011 DDI [51]
	Alprazolam							
-	0.5 mg po tab (D0)	-	8	18–38	-	-	test	Boulenc 2016 Control [52]
200 mg po tab bid (D1–D6)	0.5 mg po tab (D4)	0	8	18–38	-	-	test	Boulenc 2016 DDI [52]
400 mg po tab qd (D1–D6)	0.5 mg po tab (D4)	0	8	18–38	-	-	test	Boulenc 2016 DDI [52]
-	1 mg po cap (D0)	-	8	18–38	-	-	test	Greenblatt 1998 Control[32]
200 mg po tab bid (D1–D3)	1 mg po cap (D3)	+1	7	21–44	-	-	training	Greenblatt 1998 DDI [32]
	Midazolam							
-	0.0003 mg po sol (D0)	-	6	18–50	-	41.67	test	Halama 2013 Control [53]
400 mg po tab qd (D1–D15)	0.0003 mg po sol (D2)	0	6	18–50	-	41.67	test	Halama 2013 DDI [53]
-	0.075 mg po sol (D0)	-	4	33 (23-55)	62 (50-78)	61.9	test	Eap 2004 Control [54]
200 mg po tab bid (D1–D4)	0.075 mg po sol (D4)	0	4	33 (23-55)	62 (50-78)	61.9	test	Eap 2004 DDI [54]
-	0.075 mg/kg po sol (D0)	-	19	38.7	73.4	52.63	test	Chung 2006 Control [55]
400 mg po tab qd (D1–D10)	0.075 mg/kg po sol (D6)	-2	19	38.7	73.4	52.63	training	Chung 2006 DDI [55]
-	0.4 mg iv bol (D0)	-	6	42.80 (28–53)	-	-	test	Krishna 2009 Control [56]
400 mg po tab qd (D1–D7)	0.4 mg iv bol (D7)	0	6	42.80 (28–53)	-	-	test	Krishna 2009 DDI [56]
-	2 mg po tab (D0)	-	8	18–38	-	-	test	Boulenc 2016 Control [52]
200 mg po tab bid (D1–D5)	2 mg po tab (D4)	0	8	18–38	-	-	test	Boulenc 2016 DDI [52]
400 mg po tab qd (D1–D5)	2 mg po tab (D4)	0	8	18–38	-	-	test	Boulenc 2016 DDI [52]
-	2 mg po tab (D0)	-	7	38.80 (21–54)	-	-	test	Stoch 2009 Control [57]

(Continued on Next Page...)

Table S3.7: Clinical study data used for ketoconazole DDI model development *(continued)*

**Drug administration**		**Dose gap [h]**	**N**	**Age [years]**	**Weight [kg]**	**Females [%]**	**Dataset**	**Reference**
Ketoconazole	Victim							
400 mg po tab qd (D1)	2 mg po tab (D1)	0	12	38.80 (21–54)	-	-	test	Stoch 2009 DDI [57]
400 mg po tab qd (D1–D2)	2 mg po tab (D2)	0	9	38.80 (21–54)	-	-	test	Stoch 2009 DDI [57]
400 mg po tab qd (D1–D5)	2 mg po tab (D5)	0	9	38.80 (21–54)	-	-	test	Stoch 2009 DDI [57]
-	2 mg po tab (D0)	-	6	42.80 (28–53)	-	-	test	Krishna 2009 Control [56]
400 mg po tab qd (D1–D7)	2 mg po tab (D6)	0	6	42.80 (28–53)	-	-	test	Krishna 2009 DDI [56]
-	2 mg iv bol (D0)	-	9	26 (19–41)	77.5	33.34	test	Tsunoda 1999 Control [58]
200 mg po tab bid (D1–D2)	2 mg iv bol (D1)	0	9	26 (19–41)	77.5	33.34	test	Tsunoda 1999 DDI [58]
-	3 mg po sol (D0)	-	6	18–50	-	41.67	test	Halama 2013 Control [53]
400 mg po tab qd (D1–D15)	3 mg po sol (D8)	0	6	18–50	-	41.67	test	Halama 2013 DDI [53]
100 mg po tab qd (D1)	5 mg po sol (D1)	0	9	24–54	56–85	22.22	test	Liu 2017 DDI [59]
200 mg po tab qd (D1)	5 mg po sol (D1)	0	9	24–54	56–85	22.22	test	Liu 2017 DDI [59]
400 mg po tab qd (D1)	5 mg po sol (D1)	0	9	24–54	56–85	22.22	test	Liu 2017 DDI [59]
400 mg po tab qd (D1)	5 mg po sol (D1)	+12	6	21–46	67–80	50.00	test	Liu 2017 DDI [59]
400 mg po tab qd (D1)	5 mg po sol (D1)	+2	6	21–46	67–80	50.00	test	Liu 2017 DDI [59]
400 mg po tab qd (D1)	5 mg po sol (D1)	0	6	21–46	67–80	50.00	test	Liu 2017 DDI [59]
400 mg po tab qd (D1)	5 mg po sol (D1)	-2	6	21–46	67–80	50.00	test	Liu 2017 DDI [59]
400 mg po tab qd (D1)	5 mg po sol (D1)	-4	6	21–46	67–80	50.00	test	Liu 2017 DDI [59]
-	6 mg po sol (D0)	-	9	26 (19–41)	77.5	33.34	test	Tsunoda 1999 Control [58]
200 mg po tab bid (D1–D2)	6 mg po sol (D1)	0	9	26 (19–41)	77.5	33.34	test	Tsunoda 1999 DDI [58]
-	7.5 mg po sol (D0)	-	9	(19-26)	(52-85)	77.78	test	Olkkolla 1994 Control [60]
400 mg po tab qd (D1–D4)	7.5 mg po sol (D4)	+1	9	(19-26)	(52-85)	77.78	test	Olkkolla 1994 DDI [60]
-	10 mg po sol (D0)	-	10	34.20	72.10	57.50	test	Lam 2003 Control [61]
200 mg po tab qd (D1–D12)	10 mg po sol (D12)	+1	10	34.20	72.10	57.50	test	Lam 2003 DDI [61]
	Triazolam							
-	0.25 mg po cap (D0)	-	8	18–38	-	-	test	Greenblatt 1998 Control[32]
200 mg po tab bid (D1–D3)	0.25 mg po cap (D3)	+1	6	21–44	-	-	test	Greenblatt 1998 DDI [32]
-	0.25 mg po tab (D0)	-	9	23.80 (20–26)	65.30 (50–86)	-	test	Varhe 1994 Control [62]
400 mg po tab qd (D1–D4)	0.25 mg po tab (D4)	+1	9	23.80 (20–26)	65.30 (50–86)	66.67	test	Varhe 1994 DDI [62]
	Digoxin							
-	0.5 mg po tab	-	10	24–34	53–115	50.00	test	Larsen 2007 Control [63]
200 mg po tab qd (D1–D4)	0.5 mg po tab (D4)	0	10	24–34	53–115	50.00	test	Larsen 2007 DDI [63]

bid: twice daily, bol: bolus injection, cap: capsule, D: day, iv: intravenous, m.d.: multiple dose, n: number of individuals studied, po: oral, qd: once daily, Route: route of administration, s.d.: single dose, seq: iv and po administration on D0 were either given sequentially with a three hour gap, sim: iv and po administration on D0 were either given simultaneously, sol: solution, tab: tablet

-: no data available. Values are means and ranges, if available.

S3.2 Ketoconazole – Drug-dependent parameters

The DDI partner models with their respective parameters were derived from literature for the victim drugs alfentanil [64], alprazolam [65], midazolam [64], triazolam [66] and digoxin [64].
For the alprazolam model, Weibull model parameters *Dissolution shape* and *Dissolution time (50%)* were adpated.

Table S3.8: Drug-dependent alprazolam parameters adapted for the ketoconazole-alprazolam DDI (fed state)

Parameter	Unit	Value	Source	Original model	Reference
Weibull Dissolution shape	-	2.09	opt.	1.12	[65]
Weibull Dissolution time (50%)	min	110.10	opt.	2.20	[65]

opt.: optimized

S3.3 Ketoconazole – DDI model evaluation

S3.3.1 Plasma concentration-time profiles (Linear)

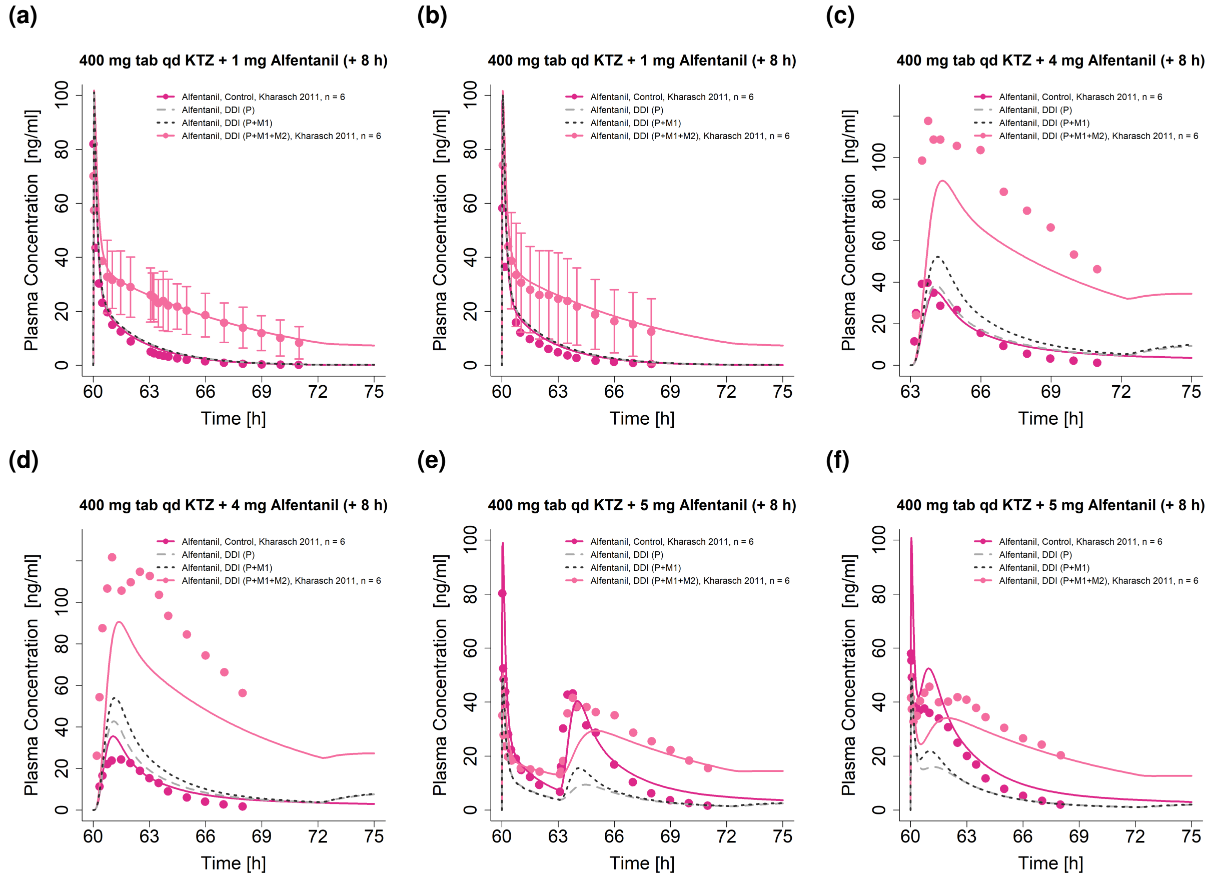

Figure S3.22: Comparison of predicted and observed ketoconazole-alfentanil DDI plasma concentration-time profiles for CYP3A4 DDIs with and without ketoconazole metabolites. Model predictions are shown as lines, observed data as dots (arithmetic mean ± SD). DDI: drug-drug interaction, KTZ: ketoconazole, M1: N-deacetylketoconazole, M2: N-hydroxy-N-deacetylketoconazole, n: number of participants, P: ketoconazole alone, qd: once daily, tab: tablet.

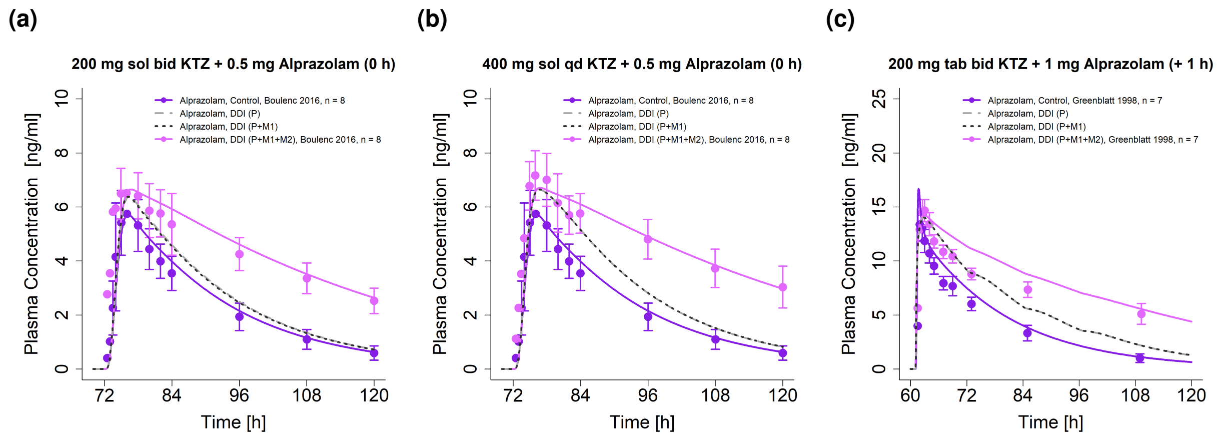

Figure S3.23: Comparison of predicted and observed ketoconazole-alprazolam DDI plasma concentration-time profiles for CYP3A4 DDIs with and without ketoconazole metabolites. Model predictions are shown as lines, observed data as dots (arithmetic mean ± SD). bid: twice daily, DDI: drug-drug interaction, KTZ: ketoconazole, M1: N-deacetylketoconazole, M2: N-hydroxy-N-deacetylketoconazole, n: number of participants, P: ketoconazole alone, qd: once daily, sol: solution, tab: tablet.

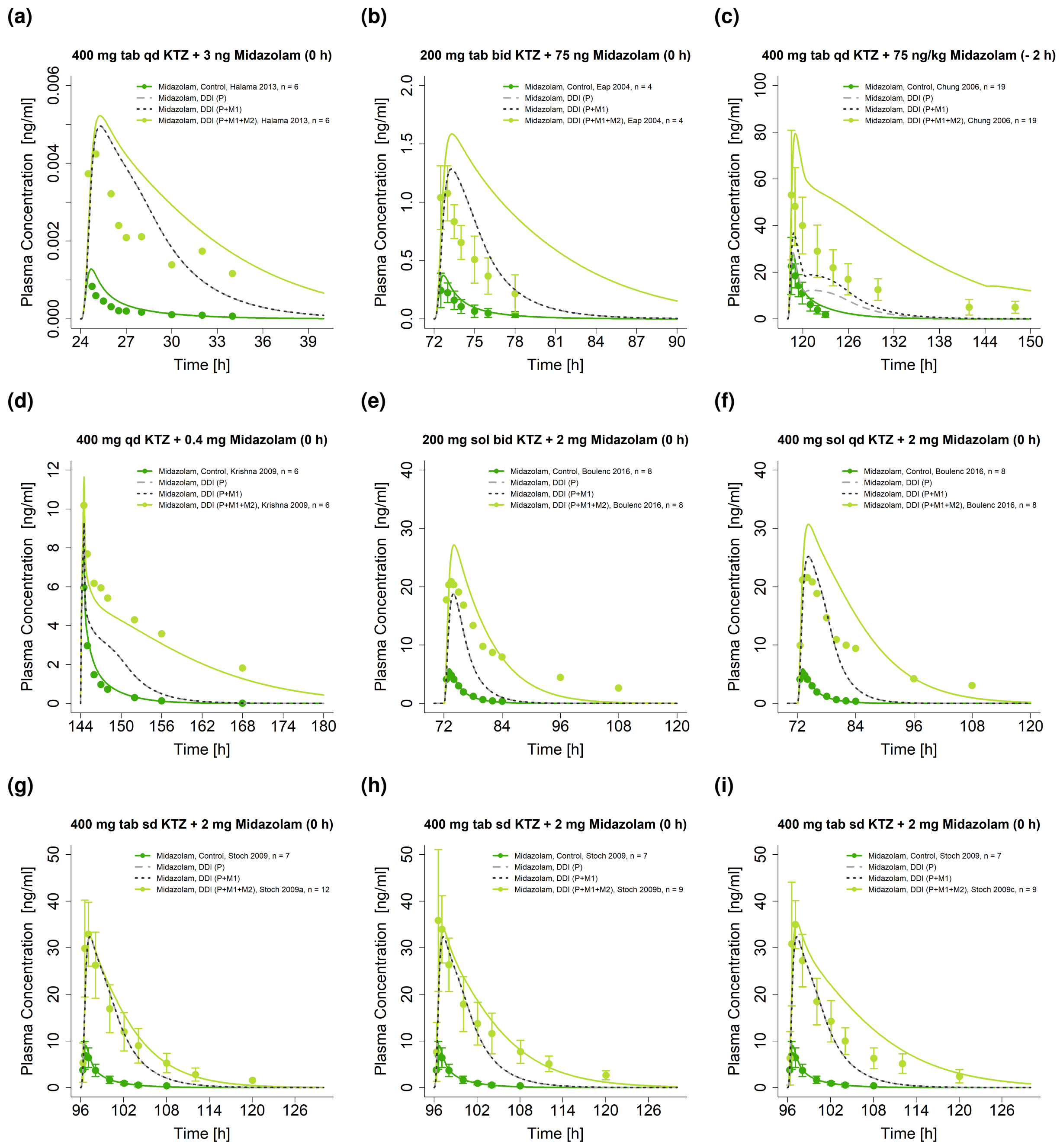

Figure S3.24: Comparison of predicted and observed ketoconazole-midazolam DDI plasma concentration-time profiles for CYP3A4 DDIs with and without ketoconazole metabolites. Model predictions are shown as lines, observed data as dots (arithmetic mean ± SD). bid: twice daily, DDI: drug-drug interaction, KTZ: ketoconazole, M1: N-deacetylketoconazole, M2: N-hydroxy-N-deacetylketoconazole, n: number of participants, P: ketoconazole alone, qd: once daily, sd: single dose, sol: solution, tab: tablet.

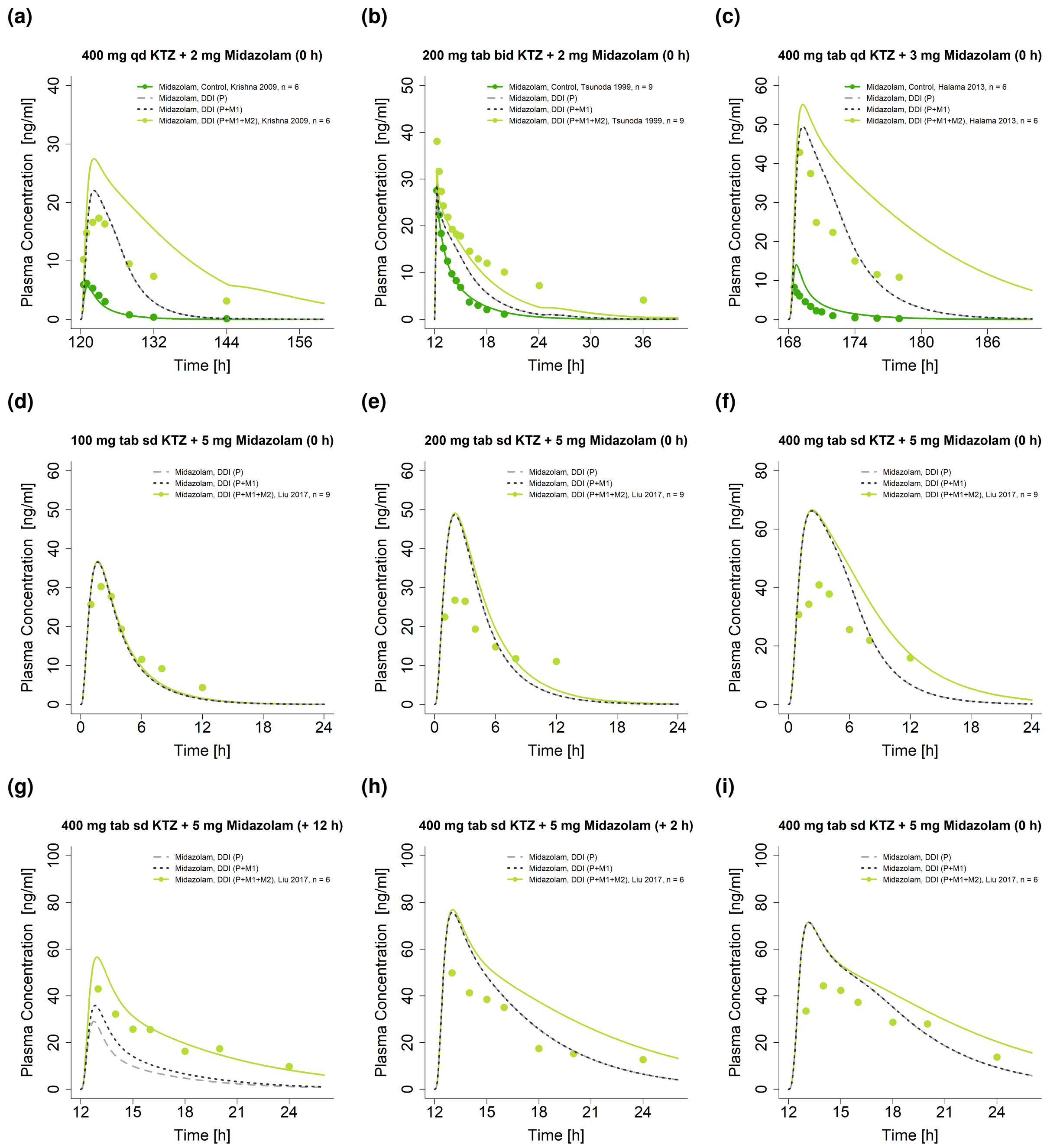

Figure S3.25: Comparison of predicted and observed ketoconazole-midazolam DDI plasma concentration-time profiles for CYP3A4 DDIs with and without ketoconazole metabolites. Model predictions are shown as lines, observed data as dots (arithmetic mean ± SD). bid: twice daily, DDI: drug-drug interaction, KTZ: ketoconazole, M1: N-deacetylketoconazole, M2: N-hydroxy-N-deacetylketoconazole, n: number of participants, P: ketoconazole alone, qd: once daily, sd: single dose, sol: solution, tab: tablet.

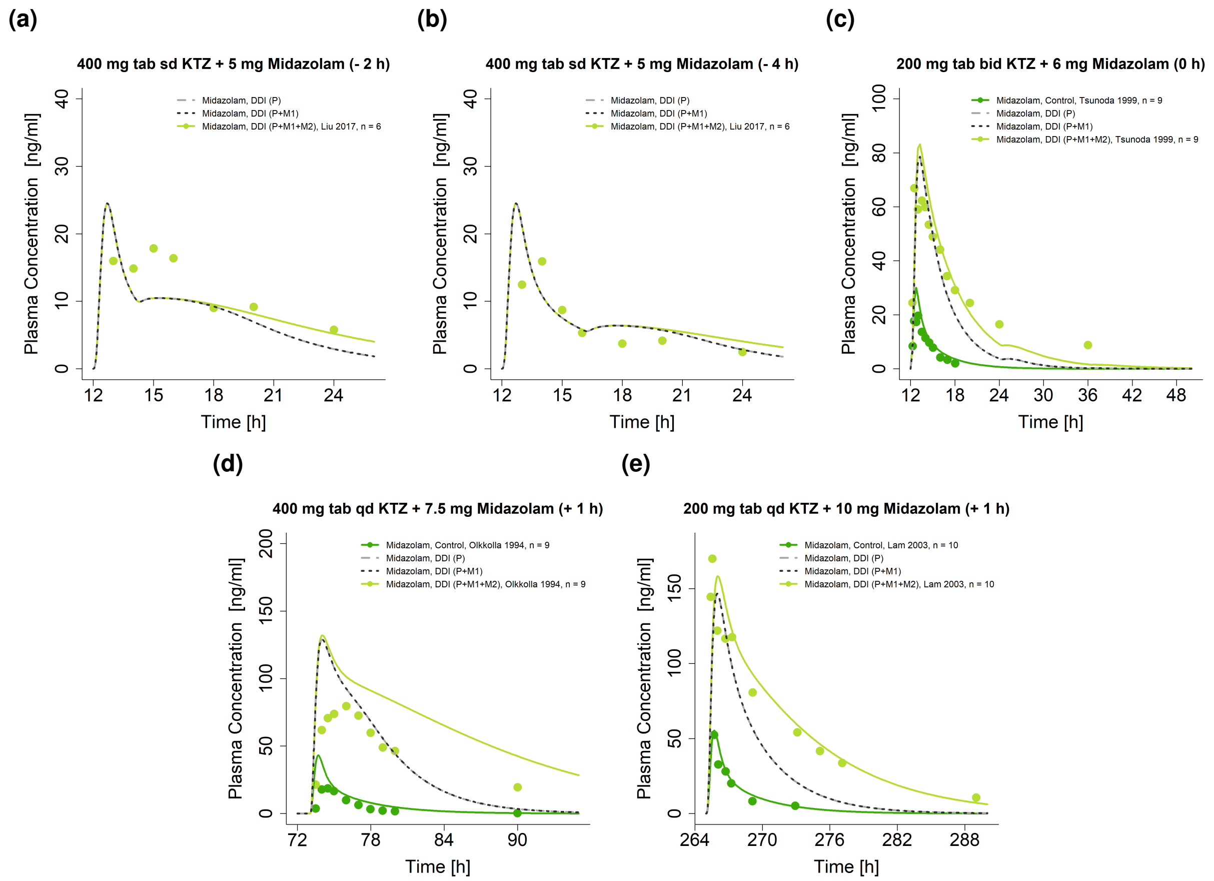

Figure S3.26: Comparison of predicted and observed ketoconazole-midazolam DDI plasma concentration-time profiles for CYP3A4 DDIs with and without ketoconazole metabolites. Model predictions are shown as lines, observed data as dots (arithmetic mean ± SD). bid: twice daily, DDI: drug-drug interaction, KTZ: ketoconazole, M1: N-deacetylketoconazole, M2: N-hydroxy-N-deacetylketoconazole, n: number of participants, P: ketoconazole alone, qd: once daily, sd: single dose, sol: solution, tab: tablet.

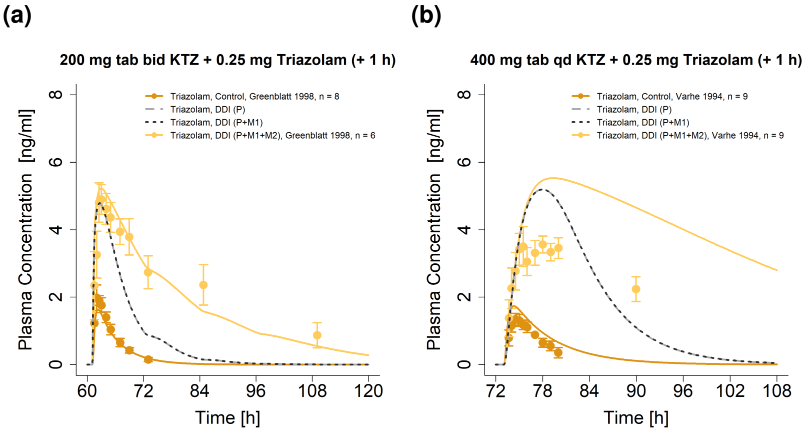

Figure S3.27: Comparison of predicted and observed ketoconazole-triazolam DDI plasma concentration-time profiles for CYP3A4 DDIs with and without ketoconazole metabolites. Model predictions are shown as lines, observed data as dots (arithmetic mean ± SD). bid: twice daily, DDI: drug-drug interaction, KTZ: ketoconazole, M1: N-deacetylketoconazole, M2: N-hydroxy-N-deacetylketoconazole, n: number of participants, P: ketoconazole alone, qd: once daily, tab: tablet.

(a)

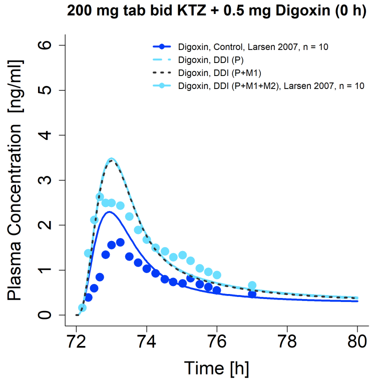

Figure S3.28: Comparison of predicted and observed ketoconazole-digoxin DDI plasma concentration-time profiles for P-gp DDIs with and without ketoconazole metabolites. Model predictions are shown as lines, observed data as dots (arithmetic mean). bid: twice daily, DDI: drug-drug interaction, KTZ: ketoconazole, M1: N-deacetylketoconazole, M2: N-hydroxy-N-deacetylketoconazole, n: number of participants, P: ketoconazole alone, tab: tablet.

S3.3.2 Plasma concentration-time profiles (Semilogarhitmic)

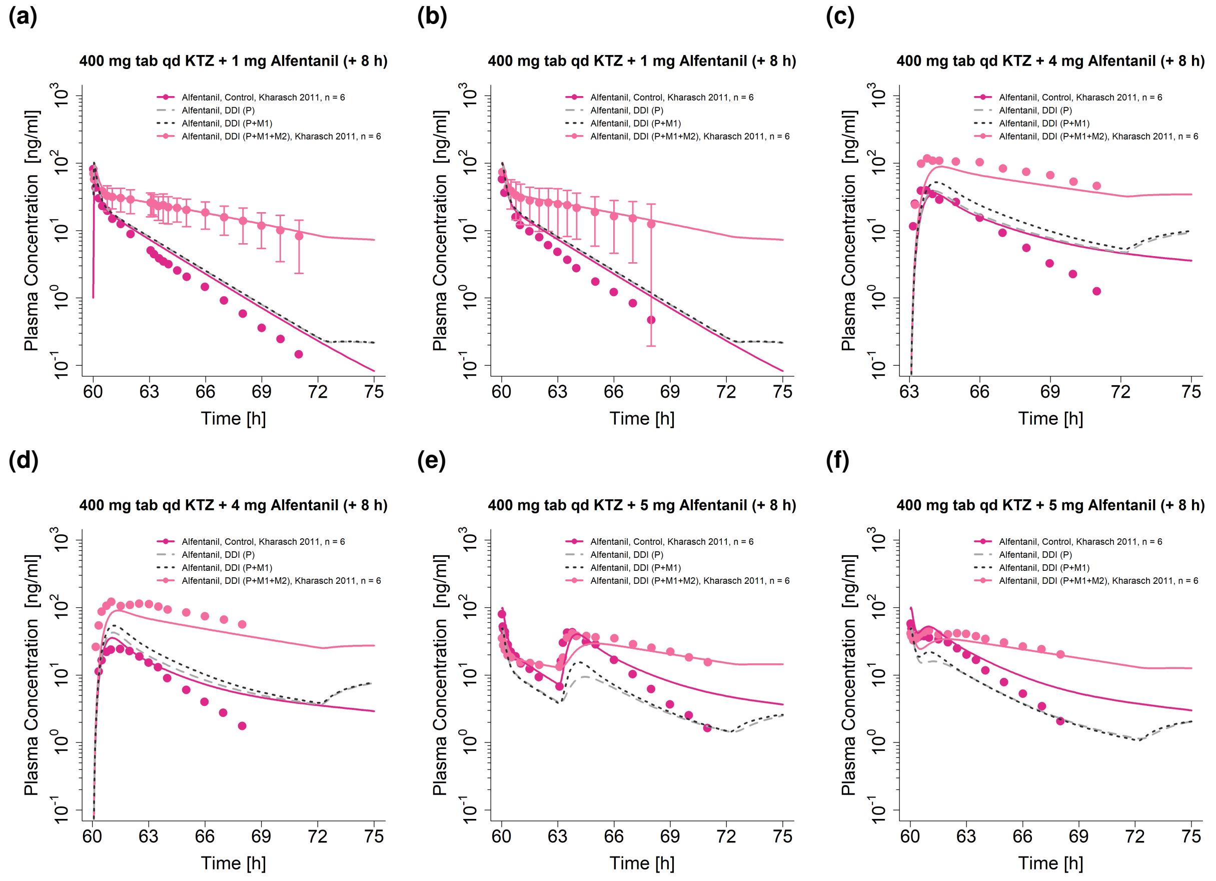

Figure S3.29: Comparison of predicted and observed ketoconazole-alfentanil DDI plasma concentration-time profiles for CYP3A4 DDIs with and without ketoconazole metabolites. Model predictions are shown as lines, observed data as dots (arithmetic mean ± SD). DDI: drug-drug interaction, KTZ: ketoconazole, M1: N-deacetylketoconazole, M2: N-hydroxy-N-deacetylketoconazole, n: number of participants, P: ketoconazole alone, qd: once daily, tab: tablet.

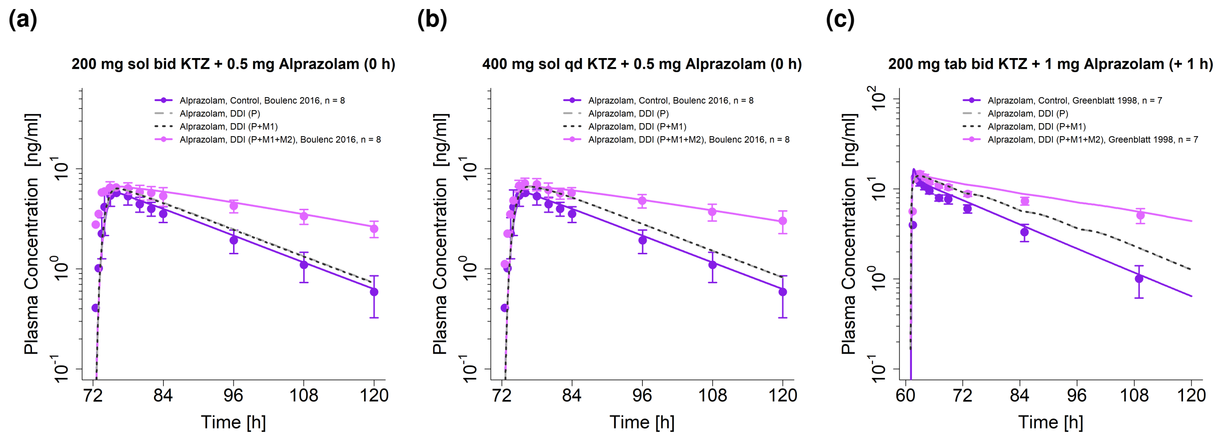

Figure S3.30: Comparison of predicted and observed ketoconazole-alprazolam DDI plasma concentration-time profiles for CYP3A4 DDIs with and without ketoconazole metabolites. Model predictions are shown as lines, observed data as dots (arithmetic mean ± SD). bid: twice daily, DDI: drug-drug interaction, KTZ: ketoconazole, M1: N-deacetylketoconazole, M2: N-hydroxy-N-deacetylketoconazole, n: number of participants, P: ketoconazole alone, qd: once daily, sol: solution, tab: tablet.

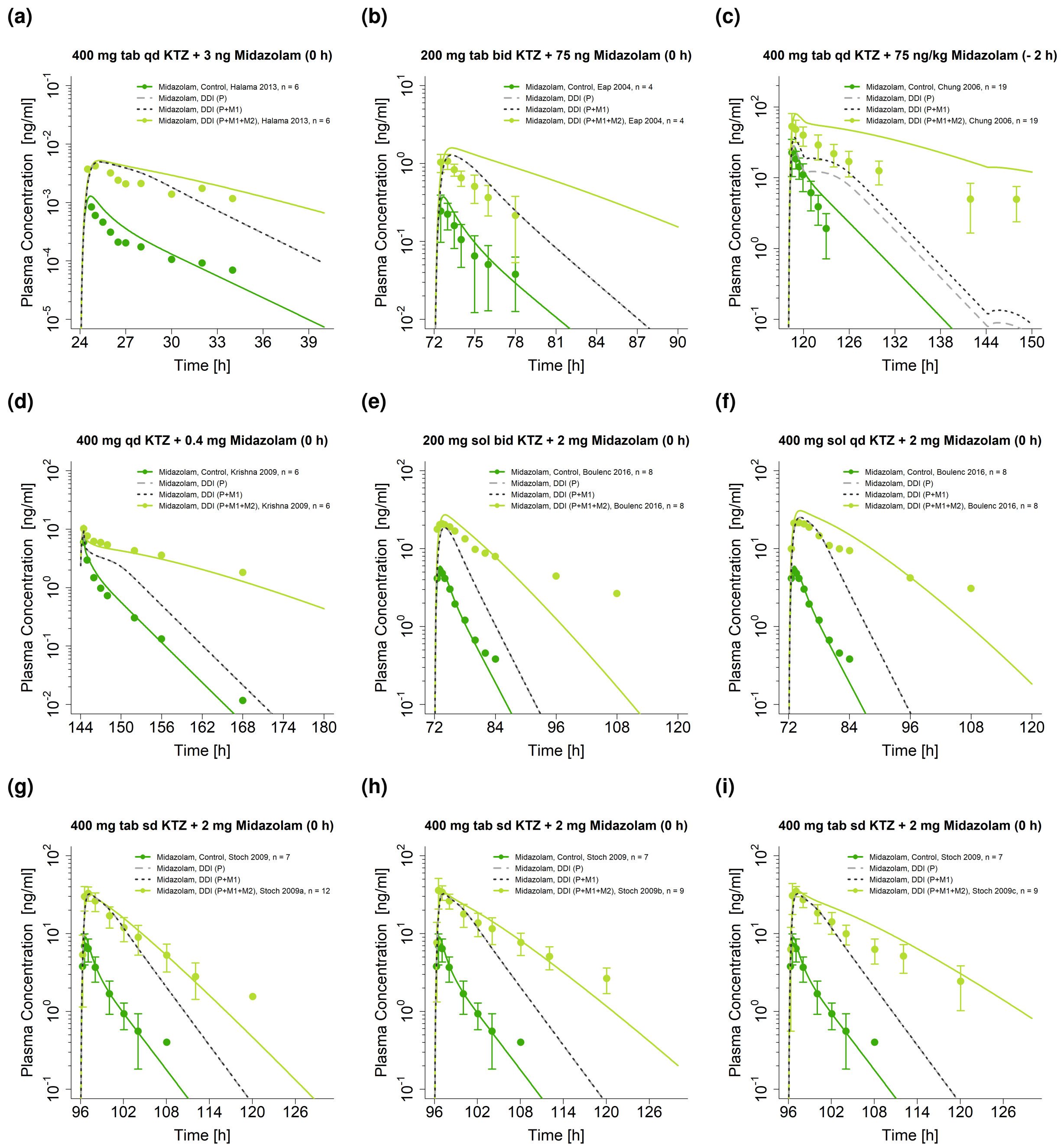

Figure S3.31: Comparison of predicted and observed ketoconazole-midazolam DDI plasma concentration-time profiles for CYP3A4 DDIs with and without ketoconazole metabolites. Model predictions are shown as lines, observed data as dots (arithmetic mean ± SD). bid: twice daily, DDI: drug-drug interaction, KTZ: ketoconazole, M1: N-deacetylketoconazole, M2: N-hydroxy-N-deacetylketoconazole, n: number of participants, P: ketoconazole alone, qd: once daily, sd: single dose, sol: solution, tab: tablet.

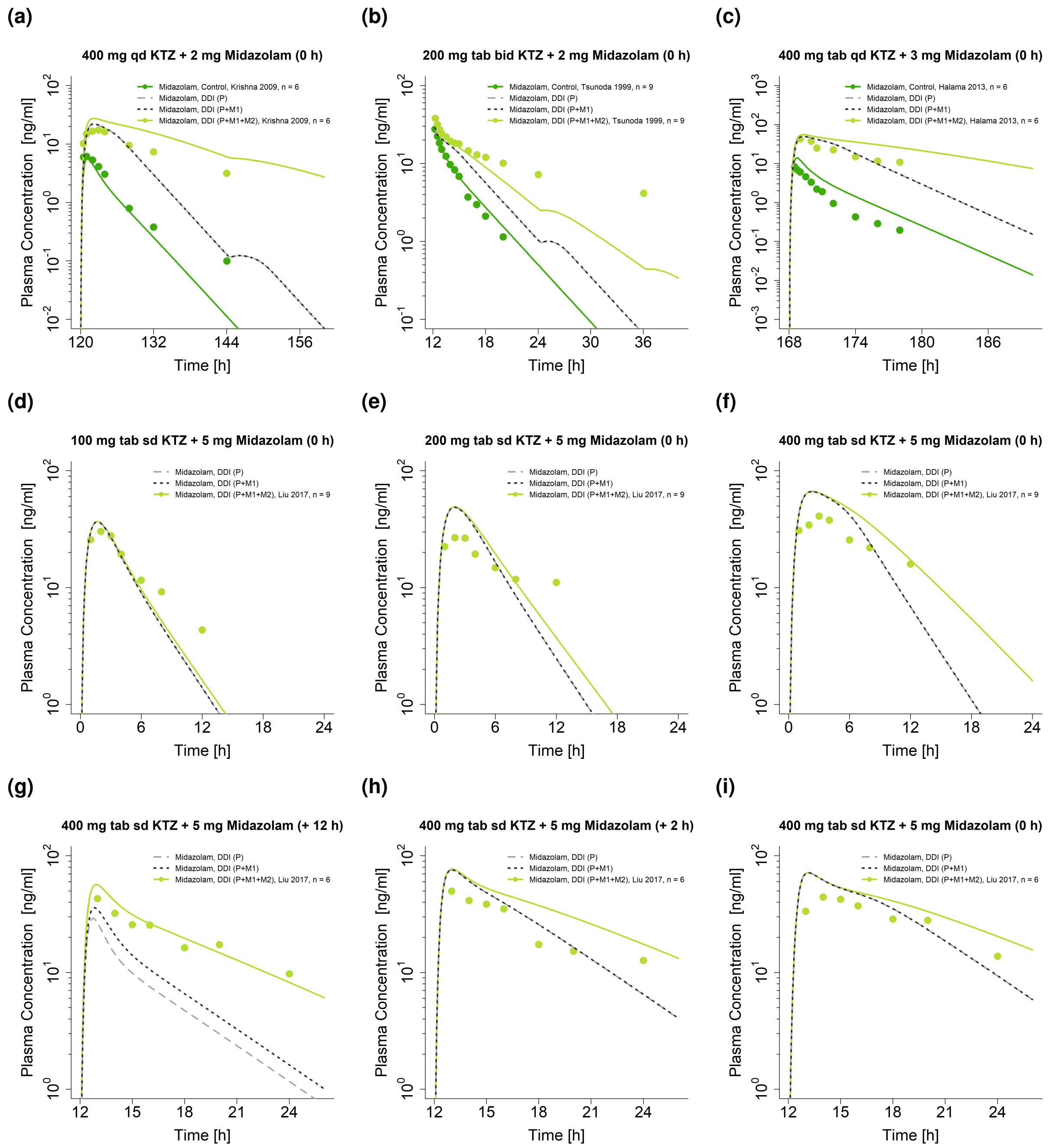

Figure S3.32: Comparison of predicted and observed ketoconazole-midazolam DDI plasma concentration-time profiles for CYP3A4 DDIs with and without ketoconazole metabolites. Model predictions are shown as lines, observed data as dots (arithmetic mean ± SD). bid: twice daily, DDI: drug-drug interaction, KTZ: ketoconazole, M1: N-deacetylketoconazole, M2: N-hydroxy-N-deacetylketoconazole, n: number of participants, P: ketoconazole alone, qd: once daily, sd: single dose, sol: solution, tab: tablet.

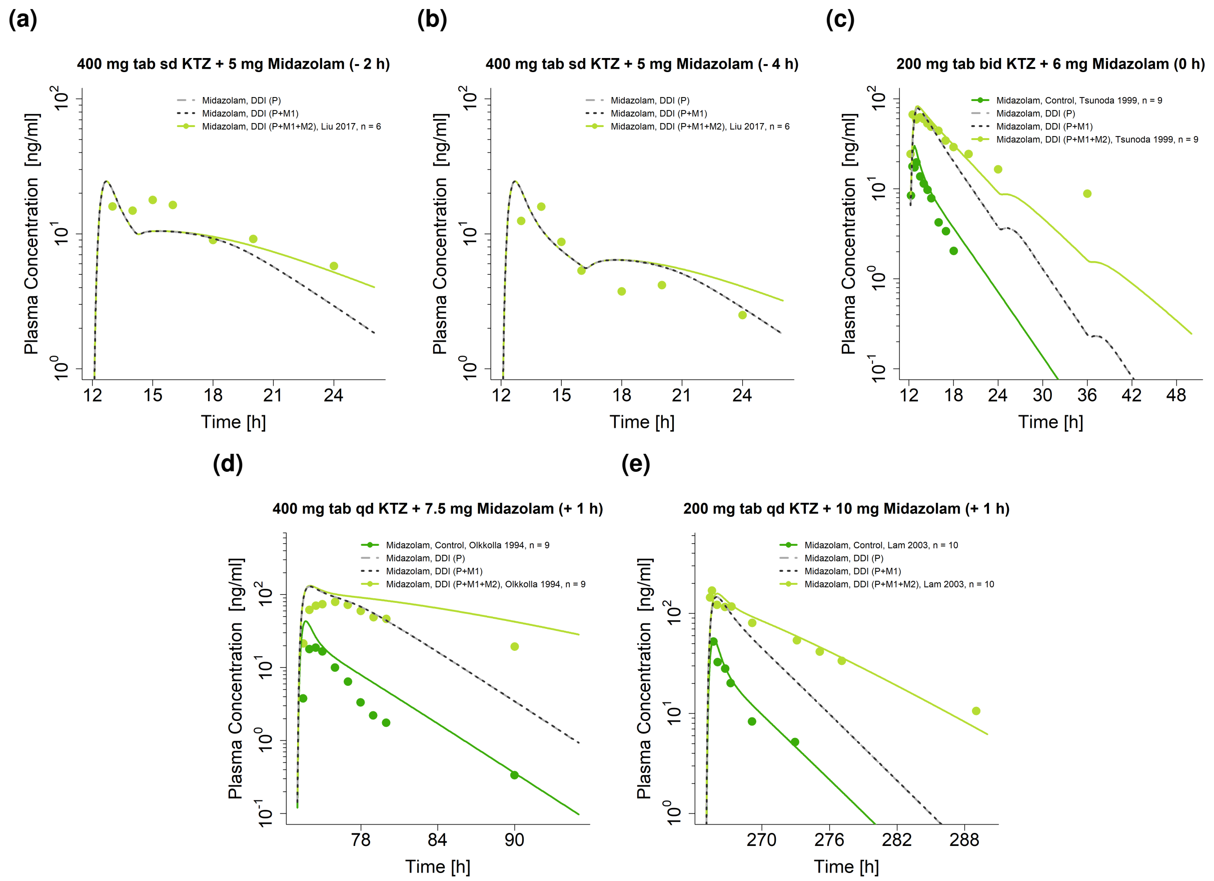

Figure S3.33: Comparison of predicted and observed ketoconazole-midazolam DDI plasma concentration-time profiles for CYP3A4 DDIs with and without ketoconazole metabolites. Model predictions are shown as lines, observed data as dots (arithmetic mean ± SD). bid: twice daily, DDI: drug-drug interaction, KTZ: ketoconazole, M1: N-deacetylketoconazole, M2: N-hydroxy-N-deacetylketoconazole, n: number of participants, P: ketoconazole alone, qd: once daily, sd: single dose, sol: solution, tab: tablet.

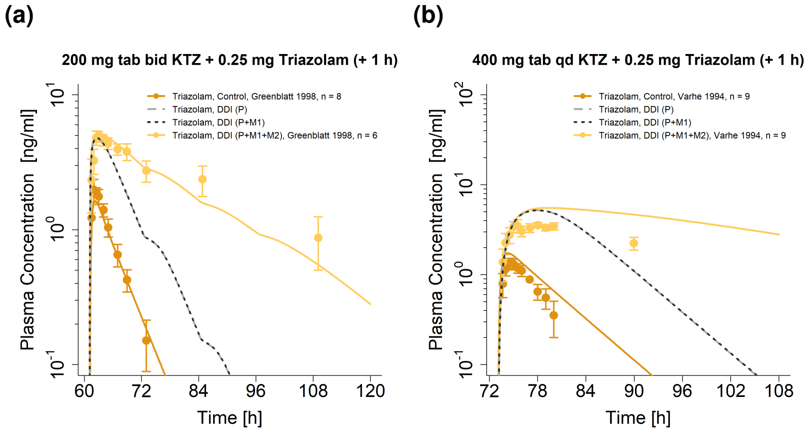

Figure S3.34: Comparison of predicted and observed ketoconazole-triazolam DDI plasma concentration-time profiles for CYP3A4 DDIs with and without ketoconazole metabolites. Model predictions are shown as lines, observed data as dots (arithmetic mean ± SD). bid: twice daily, DDI: drug-drug interaction, KTZ: ketoconazole, M1: N-deacetylketoconazole, M2: N-hydroxy-N-deacetylketoconazole, n: number of participants, P: ketoconazole alone, qd: once daily, tab: tablet.

(a)

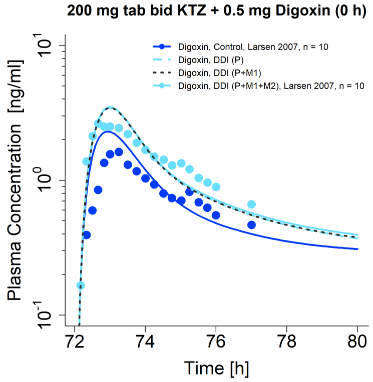

Figure S3.35: Comparison of predicted and observed ketoconazole-digoxin DDI plasma concentration-time profiles for P-gp DDIs with and without ketoconazole metabolites. Model predictions are shown as lines, observed data as dots (arithmetic mean ± SD). bid: twice daily, DDI: drug-drug interaction, KTZ: ketoconazole, M1: N-deacetylketoconazole, M2: N-hydroxy-N-deacetylketoconazole, n: number of participants, P: ketoconazole alone, tab: tablet.

S3.3.3 DDI AUC_{last} and DDI C_{max} goodness-of-fit plots

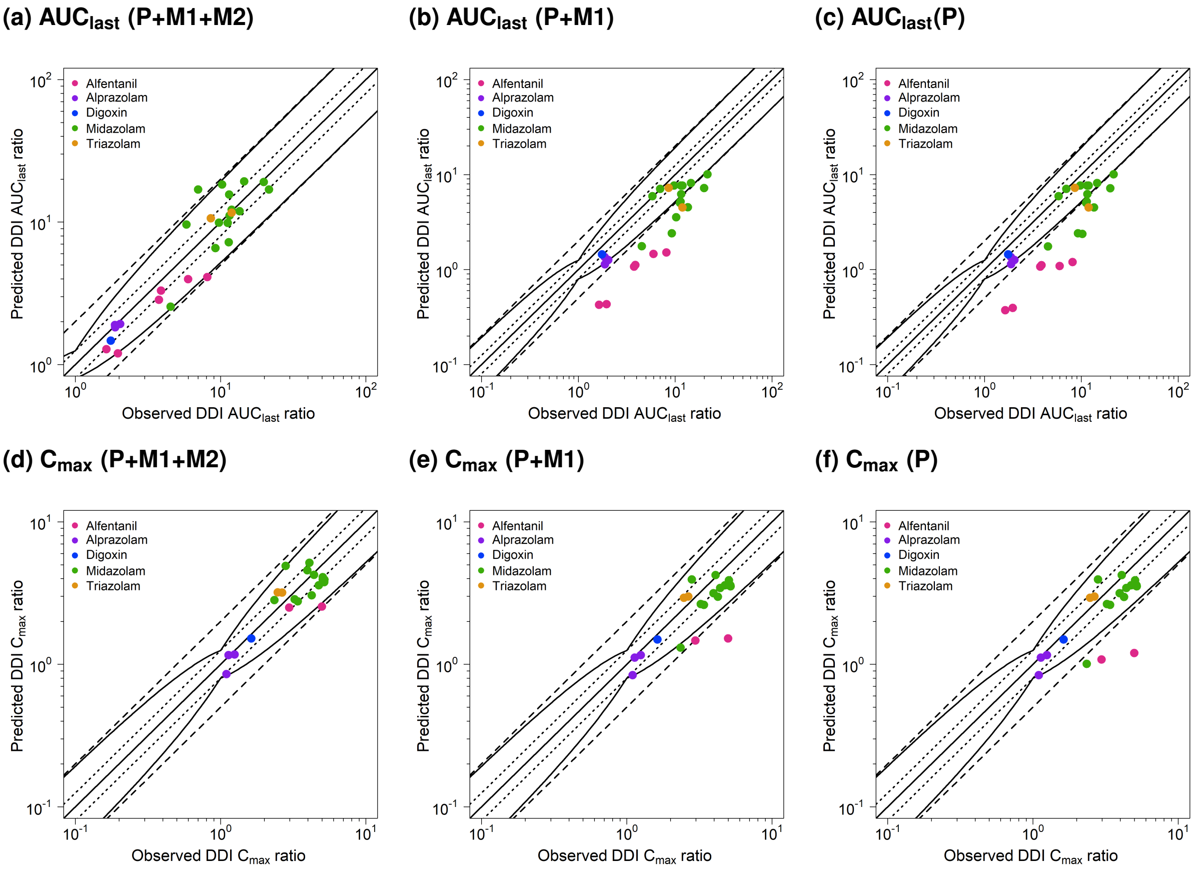

Figure S3.36: Predicted compared to observed victim drug DDI AUC_{last} and DDI C_{max} ratios for ketoconazole DDIs with and without its metabolites. The straight solid line marks the line of identity. The curved solid lines show the prediction acceptance limits proposed by Guest et al. including 1.25-fold variability [50]. Dotted lines indicate 1.25-fold, dashed lines indicate 2-fold deviation. AUC_{last}: area under the plasma concentration-time curve from the time of drug administration to the last concentration measurement, C_{max}: maximum plasma concentration, DDI: drug-drug interaction, M1: N-deacetylketoconazole, M2: N-hydroxy-N-deacetylketoconazole, P: ketoconazole alone.

S3.3.4 Geometric mean fold error of predicted DDI AUC_{last} and DDI C_{max} ratios

Table S3.9: Predicted and observed DDI AUC_{last} and C_{max} ratios of the victim drugs during DDIs, simulated with and without ketoconazole metabolite involvement

Drug administration			N	DDI AUC_{last} ratio			DDI C_{max} ratio			Reference
Ketoconazole	Victim	Gap [h]		Pred	Obs	Pred/Obs	Pred	Obs	Pred/Obs	
	Alfentanil									
400 mg po tab qd (D1–D4)	0.5 mg iv bol seq (D5)	+8	6	3.31	3.88	0.85	-	-	-	Kharash 2011 DDI (P+M1+M2) [51]
400 mg po tab qd (D1–D4)	0.5 mg iv bol seq (D5)	+8	6	1.12	3.88	0.29	-	-	-	Kharash 2011 DDI (P+M1) [51]
400 mg po tab qd (D1–D4)	0.5 mg iv bol seq (D5)	+8	6	1.12	3.88	0.29	-	-	-	Kharash 2011 DDI (P) [51]
400 mg po tab qd (D1–D4)	0.5 mg iv bol sim (D5)	+8	6	2.85	3.76	0.76	-	-	-	Kharash 2011 DDI (P+M1+M2) [51]
400 mg po tab qd (D1–D4)	0.5 mg iv bol sim (D5)	+8	6	1.08	3.76	0.29	-	-	-	Kharash 2011 DDI (P+M1) [51]
400 mg po tab qd (D1–D4)	0.5 mg iv bol sim (D5)	+8	6	1.07	3.76	0.29	-	-	-	Kharash 2011 DDI (P) [51]
400 mg po tab qd (D1–D4)	1 mg po tab seq (D5)	+8	6	3.98	5.97	0.67	2.50	2.96	0.84	Kharash 2011 DDI (P+M1+M2) [51]
400 mg po tab qd (D1–D4)	1 mg po tab seq (D5)	+8	6	1.46	5.97	0.25	1.47	2.96	0.50	Kharash 2011 DDI (P+M1) [51]
400 mg po tab qd (D1–D4)	1 mg po tab seq (D5)	+8	6	1.09	5.97	0.18	1.08	2.96	0.37	Kharash 2011 DDI (P) [51]
400 mg po tab qd (D1–D4)	1 mg po tab sim (D5)	+8	6	4.12	8.11	0.51	2.55	4.99	0.51	Kharash 2011 DDI (P+M1+M2) [51]
400 mg po tab qd (D1–D4)	1 mg po tab sim (D5)	+8	6	1.52	8.11	0.19	1.52	4.99	0.30	Kharash 2011 DDI (P+M1) [51]
400 mg po tab qd (D1–D4)	1 mg po tab sim (D5)	+8	6	1.20	8.11	0.15	1.20	4.99	0.24	Kharash 2011 DDI (P) [51]
400 mg po tab qd (D1–D4)	0.5 bol & 1 mg tab seq (D5)	+8	6	1.28	1.63	0.78	-	-	-	Kharash 2011 DDI (P+M1+M2) [51]
400 mg po tab qd (D1–D4)	0.5 bol & 1 mg tab seq (D5)	+8	6	0.43	1.63	0.26	-	-	-	Kharash 2011 DDI (P+M1) [51]
400 mg po tab qd (D1–D4)	0.5 bol & 1 mg tab seq (D5)	+8	6	0.37	1.63	0.23	-	-	-	Kharash 2011 DDI (P) [51]
400 mg po tab qd (D1–D4)	0.5 bol & 1 mg tab seq (D5)	+8	6	1.20	1.96	0.61	-	-	-	Kharash 2011 DDI (P+M1+M2) [51]
400 mg po tab qd (D1–D4)	0.5 bol & 1 mg tab seq (D5)	+8	6	0.43	1.96	0.22	-	-	-	Kharash 2011 DDI (P+M1) [51]
400 mg po tab qd (D1–D4)	0.5 bol & 1 mg tab seq (D5)	+8	6	0.40	1.96	0.20	-	-	-	Kharash 2011 DDI (P) [51]
	Mean GMFE DDI (P+M1+M2)					1.48			1.57	
	Mean GMFE DDI (P+M1)					4.12			2.65	
	Mean GMFE DDI (P)					4.76			3.44	
	Alprazolam									
200 mg po tab bid (D1–D6)	0.5 mg po tab (D4)	0	8	1.93	2.03	0.95	1.17	1.25	0.94	Boulenc 2016 DDI (P+M1+M2) [52]
200 mg po tab bid (D1–D6)	0.5 mg po tab (D4)	0	8	1.27	2.03	0.62	1.16	1.25	0.93	Boulenc 2016 DDI (P+M1) [52]
200 mg po tab bid (D1–D6)	0.5 mg po tab (D4)	0	8	1.26	2.03	0.62	1.16	1.25	0.93	Boulenc 2016 DDI (P) [52]
400 mg po tab qd (D1–D6)	0.5 mg po tab (D4)	0	8	1.83	1.88	0.97	1.16	1.13	1.02	Boulenc 2016 DDI (P+M1+M2) [52]

(Continued on next page...)

Table S3.9: Predicted and observed DDI AUC_{last} and C_{max} ratios of the victim drugs during DDIs, simulated with and without ketoconazole metabolite involvement *(continued)*

**Drug administration**			**N**	**DDI AUC_{last} ratio**			**DDI C_{max} ratio**			**Reference**
Ketoconazole	Victim	Gap [h]		Pred	Obs	Pred/Obs	Pred	Obs	Pred/Obs	
400 mg po tab qd (D1–D6)	0.5 mg po tab (D4)	0	8	1.14	1.88	0.61	1.12	1.13	0.98	Boulenc 2016 DDI (P+M1) [52]
400 mg po tab qd (D1–D6)	0.5 mg po tab (D4)	0	8	1.14	1.88	0.61	1.12	1.13	0.98	Boulenc 2016 DDI (P) [52]
200 mg po tab bid (D1–D3)	1 mg po cap (D3)	+1	7	1.90	1.87	1.01	0.85	1.10	0.78	Greenblatt 1998 DDI (P+M1+M2) [32]
200 mg po tab bid (D1–D3)	1 mg po cap (D3)	+1	7	1.36	1.87	0.72	0.84	1.10	0.77	Greenblatt 1998 DDI (P+M1) [32]
200 mg po tab bid (D1–D3)	1 mg po cap (D3)	+1	7	1.35	1.87	0.72	0.84	1.10	0.77	Greenblatt 1998 DDI (P) [32]
		Mean GMFE DDI (P+M1+M2)				1.03			1.12	
		Mean GMFE DDI (P+M1)				1.55			1.13	
		Mean GMFE DDI (P)				1.54			1.13	
	Midazolam									
400 mg po tab qd (D1–D15)	0.0003 mg po sol (D2)	0	6	16.91	21.56	0.78	4.09	5.06	0.81	Halama 2013 DDI (P+M1+M2) [53]
400 mg po tab qd (D1–D15)	0.0003 mg po sol (D2)	0	6	10.08	21.56	0.47	3.89	5.06	0.77	Halama 2013 DDI (P+M1) [53]
400 mg po tab qd (D1–D15)	0.0003 mg po sol (D2)	0	6	10.07	21.56	0.47	3.89	5.06	0.77	Halama 2013 DDI (P) [53]
200 mg po tab bid (D1–D4)	0.075 mg po sol (D4)	0	4	9.63	5.82	1.65	4.25	4.39	0.97	Eap 2004 DDI (P+M1+M2) [54]
200 mg po tab bid (D1–D4)	0.075 mg po sol (D4)	0	4	5.94	5.82	1.02	3.44	4.39	0.78	Eap 2004 DDI (P+M1) [54]
200 mg po tab bid (D1–D4)	0.075 mg po sol (D4)	0	4	5.93	5.82	1.02	3.44	4.39	0.78	Eap 2004 DDI (P) [54]
400 mg po tab qd (D1–D10)	0.075 mg/kg po sol (D6)	-2	19	18.39	10.24	1.80	2.82	2.34	1.20	Chung 2006 DDI (P+M1+M2) [55]
400 mg po tab qd (D1–D10)	0.075 mg/kg po sol (D6)	-2	19	3.56	10.24	0.35	1.31	2.34	0.56	Chung 2006 DDI (P+M1) [55]
400 mg po tab qd (D1–D10)	0.075 mg/kg po sol (D6)	-2	19	2.38	10.24	0.23	1.01	2.34	0.43	Chung 2006 DDI (P) [55]
400 mg po tab qd (D1–D7)	0.4 mg iv bol (D7)	0	6	6.57	9.21	0.71	-	-	-	Krishna 2009 DDI (P+M1+M2) [56]
400 mg po tab qd (D1–D7)	0.4 mg iv bol (D7)	0	6	2.41	9.21	0.26	-	-	-	Krishna 2009 DDI (P+M1) [56]
400 mg po tab qd (D1–D7)	0.4 mg iv bol (D7)	0	6	2.41	9.21	0.26	-	-	-	Krishna 2009 DDI (P) [56]
200 mg po tab bid (D1–D5)	2 mg po tab (D4)	0	8	19.30	14.55	1.33	5.17	4.08	1.27	Boulenc 2016 DDI (P+M1+M2) [52]
200 mg po tab bid (D1–D5)	2 mg po tab (D4)	0	8	8.15	14.55	0.56	4.24	4.08	1.04	Boulenc 2016 DDI (P+M1) [52]
200 mg po tab bid (D1–D5)	2 mg po tab (D4)	0	8	8.14	14.55	0.56	4.24	4.08	1.04	Boulenc 2016 DDI (P) [52]
400 mg po tab qd (D1–D5)	2 mg po tab (D4)	0	8	11.94	13.51	0.88	4.57	3.96	1.16	Boulenc 2016 DDI (P+M1+M2) [52]
400 mg po tab qd (D1–D5)	2 mg po tab (D4)	0	8	4.53	13.51	0.34	3.16	3.96	0.80	Boulenc 2016 DDI (P+M1) [52]
400 mg po tab qd (D1–D5)	2 mg po tab (D4)	0	8	4.52	13.51	0.33	3.15	3.96	0.80	Boulenc 2016 DDI (P) [52]
400 mg po tab qd (D1)	2 mg po tab (D1)	0	12	9.91	9.75	1.02	3.59	4.75	0.76	Stoch 2009 DDI (P+M1+M2) [57]
400 mg po tab qd (D1)	2 mg po tab (D1)	0	12	7.67	9.75	0.79	3.59	4.75	0.76	Stoch 2009 DDI (P+M1) [57]

(Continued on next page...)

Table S3.9: Predicted and observed DDI AUC_{last} and C_{max} ratios of the victim drugs during DDIs, simulated with and without ketoconazole metabolite involvement *(continued)*

**Drug administration**			**N**	**DDI AUC_{last} ratio**			**DDI C_{max} ratio**			**Reference**
Ketoconazole	Victim	Gap [h]		Pred	Obs	Pred/Obs	Pred	Obs	Pred/Obs	
400 mg po tab qd (D1)	2 mg po tab (D1)	0	12	7.66	9.75	0.79	3.58	4.75	0.76	Stoch 2009 DDI (P) [57]
400 mg po tab qd (D1–D2)	2 mg po tab (D2)	0	9	12.21	11.90	1.03	3.77	5.17	0.73	Stoch 2009 DDI (P+M1+M2) [57]
400 mg po tab qd (D1–D2)	2 mg po tab (D2)	0	9	7.67	11.90	0.64	3.59	5.17	0.69	Stoch 2009 DDI (P+M1) [57]
400 mg po tab qd (D1–D2)	2 mg po tab (D2)	0	9	7.67	11.90	0.64	3.59	5.17	0.69	Stoch 2009 DDI (P) [57]
400 mg po tab qd (D1–D5)	2 mg po tab (D5)	0	9	15.63	11.46	1.36	3.94	5.04	0.78	Stoch 2009 DDI (P+M1+M2) [57]
400 mg po tab qd (D1–D5)	2 mg po tab (D5)	0	9	7.68	11.46	0.67	3.59	5.04	0.71	Stoch 2009 DDI (P+M1) [57]
400 mg po tab qd (D1–D5)	2 mg po tab (D5)	0	9	7.67	11.46	0.67	3.59	5.04	0.71	Stoch 2009 DDI (P) [57]
400 mg po tab qd (D1–D7)	2 mg po tab (D6)	0	6	16.94	7.01	2.42	4.92	2.81	1.75	Krishna 2009 DDI (P+M1+M2) [56]
400 mg po tab qd (D1–D7)	2 mg po tab (D6)	0	6	7.09	7.01	1.01	3.95	2.81	1.41	Krishna 2009 DDI (P+M1) [56]
400 mg po tab qd (D1–D7)	2 mg po tab (D6)	0	6	7.09	7.01	1.01	3.95	2.81	1.41	Krishna 2009 DDI (P) [56]
200 mg po tab bid (D1–D2)	2 mg iv bol (D1)	0	9	2.55	4.53	0.56	-	-	-	Tsunoda 1999 DDI (P+M1+M2) [58]
200 mg po tab bid (D1–D2)	2 mg iv bol (D1)	0	9	1.76	4.53	0.39	-	-	-	Tsunoda 1999 DDI (P+M1) [58]
200 mg po tab bid (D1–D2)	2 mg iv bol (D1)	0	9	1.76	4.53	0.39	-	-	-	Tsunoda 1999 DDI (P) [58]
400 mg po tab qd (D1–D15)	3 mg po sol (D8)	0	6	19.14	19.85	0.96	3.94	5.18	0.76	Halama 2013 DDI (P+M1+M2) [53]
400 mg po tab qd (D1–D15)	3 mg po sol (D8)	0	6	7.21	19.85	0.36	3.54	5.18	0.68	Halama 2013 DDI (P+M1) [53]
400 mg po tab qd (D1–D15)	3 mg po sol (D8)	0	6	7.21	19.85	0.36	3.54	5.18	0.68	Halama 2013 DDI (P) [53]
200 mg po tab bid (D1–D2)	6 mg po sol (D1)	0	9	7.23	11.39	0.63	2.77	3.40	0.81	Tsunoda 1999 DDI (P+M1+M2) [58]
200 mg po tab bid (D1–D2)	6 mg po sol (D1)	0	9	5.21	11.39	0.46	2.62	3.40	0.77	Tsunoda 1999 DDI (P+M1) [58]
200 mg po tab bid (D1–D2)	6 mg po sol (D1)	0	9	5.21	11.39	0.46	2.62	3.40	0.77	Tsunoda 1999 DDI (P) [58]
400 mg po tab qd (D1–D4)	7.5 mg po sol (D4)	+1	9	11.12	11.53	0.96	3.05	4.24	0.72	Olkkolla 1994 DDI (P+M1+M2) [60]
400 mg po tab qd (D1–D4)	7.5 mg po sol (D4)	+1	9	6.24	11.53	0.54	2.98	4.24	0.70	Olkkolla 1994 DDI (P+M1) [60]
400 mg po tab qd (D1–D4)	7.5 mg po sol (D4)	+1	9	6.23	11.53	0.54	2.98	4.24	0.70	Olkkolla 1994 DDI (P) [60]
200 mg po tab qd (D1–D12)	10 mg po sol (D12)	+1	10	9.87	11.18	0.88	2.86	3.23	0.88	Lam 2003 DDI (P+M1+M2) [61]
200 mg po tab qd (D1–D12)	10 mg po sol (D12)	+1	10	5.05	11.18	0.45	2.65	3.23	0.82	Lam 2003 DDI (P+M1) [61]
200 mg po tab qd (D1–D12)	10 mg po sol (D12)	+1	10	5.05	11.18	0.45	2.65	3.23	0.82	Lam 2003 DDI (P) [61]
		Mean GMFE DDI(P+M1+M2)				1.40			1.30	
		Mean GMFE DDI (P+M1)				2.10			1.36	
		Mean GMFE DDI (P)				2.20			1.38	

(Continued on next page...)

Table S3.9: Predicted and observed DDI AUC_{last} and C_{max} ratios of the victim drugs during DDIs, simulated with and without ketoconazole metabolite involvement *(continued)*

Drug administration			N	DDI AUC_{last} ratio			DDI C_{max} ratio			Reference
Ketoconazole	Victim	Gap [h]		Pred	Obs	Pred/Obs	Pred	Obs	Pred/Obs	
	Triazolam									
200 mg po tab bid (D1–D3)	0.25 mg po cap (D3)	+1	6	11.67	11.90	0.98	3.20	2.47	1.29	Greenblatt 1998 DDI (P+M1+M2) [32]
200 mg po tab bid (D1–D3)	0.25 mg po cap (D3)	+1	6	4.52	11.90	0.38	2.93	2.47	1.19	Greenblatt 1998 DDI (P+M1) [32]
200 mg po tab bid (D1–D3)	0.25 mg po cap (D3)	+1	6	4.51	11.90	0.38	2.93	2.47	1.19	Greenblatt 1998 DDI (P) [32]
400 mg po tab qd (D1–D4)	0.25 mg po tab (D4)	+1	9	10.64	8.56	1.24	3.19	2.65	1.20	Varhe 1994 DDI (P+M1+M2)[62]
400 mg po tab qd (D1–D4)	0.25 mg po tab (D4)	+1	9	7.27	8.56	0.85	3.00	2.65	1.13	Varhe 1994 DDI (P+M1) [62]
400 mg po tab qd (D1–D4)	0.25 mg po tab (D4)	+1	9	7.26	8.56	0.85	3.00	2.65	1.13	Varhe 1994 DDI (P) [62]
	Mean GMFE DDI (P+M1+M2)					1.13			1.25	
	Mean GMFE DDI (P+M1)					1.91			1.16	
	Mean GMFE DDI (P)					1.91			1.16	
	Digoxin									
200 mg po tab qd (D1–D4)	0.5 mg po tab (D4)	0	10	1.47	1.75	0.84	1.52	1.63	0.93	Larsen 2007 DDI (P+M1+M2) [63]
200 mg po tab qd (D1–D4)	0.5 mg po tab (D4)	0	10	1.45	1.75	0.83	1.50	1.63	0.92	Larsen 2007 DDI (P+M1) [63]
200 mg po tab qd (D1–D4)	0.5 mg po tab (D4)	0	10	1.45	1.75	0.83	1.50	1.63	0.92	Larsen 2007 DDI (P) [63]
	Mean GMFE DDI(P+M1+M2)					1.19			1.07	
	Mean GMFE DDI (P+M1)					1.21			1.09	
	Mean GMFE DDI (P)					1.21			1.09	
	**Overall mean GMFE (range) DDI (P+M1+M2)**					**1.35 (1.01–2.41)**			**1.27 (1.02–1.96)**	
	**pred/obs DDI ratios within two-fold**					**96.30% (26/27) $\leq$ 2**			**100.00% (21/21) $\leq$ 2**	
	**Overall mean GMFE (range) DDI (P+M1)**					**2.44 (1.01–5.34)**			**1.42 (1.02–3.28)**	
	**pred/obs DDI ratios within two-fold**					**44.45% (12/27) $\leq$ 2**			**90.48% (19/21) $\leq$ 2**	
	**Overall mean GMFE (range) DDI (P)**					**2.64 (1.01–6.75)**			**1.52 (1.02–4.15)**	
	**pred/obs DDI ratios within two-fold**					**44.45% (12/27) $\leq$ 2**			**85.71% (18/21) $\leq$ 2**	

AUC_{last}: area under the plasma concentration-time curve calculated from the first to last time point of measurement, bid: twice daily, cap: capsule, C_{max}: maximum plasma concentration, DDI: drug-drug-interaction, GMFE: geometric mean fold error, iv: intravenous, M1: N-deacetylketoconazole, M2: N-hydroxy-N-deacetylketoconazle, N: number of individuals studied, obs: observed, pred: predicted, P: ketoconazole alone, seq: iv and po administration on D0 were either given sequentially with a three hour gap, sim: iv and po administration on D0 were either given simultaneously, sol: solution, tab: tablet

References

[1] M. Meyer, S. Schneckener, B. Ludewig, L. Kuepfer, and J. Lippert. Using expression data for quantification of active processes in physiologically based pharmacokinetic modeling. *Drug metabolism and disposition: the biological fate of chemicals*, 40(5):892–901, may 2012. doi: 10.1124/dmd.111.043174.

[2] M. Nishimura and S. Naito. Tissue-specific mRNA expression profiles of human phase I metabolizing enzymes except for cytochrome P450 and phase II metabolizing enzymes. *Drug metabolism and pharmacokinetics*, 21(5): 357–74, 2006. doi: 10.2133/dmpk.21.357.

[3] A. D. Rodrigues. Integrated cytochrome P450 reaction phenotyping: attempting to bridge the gap between cDNA-expressed cytochromes P450 and native human liver microsomes. *Biochemical pharmacology*, 57(5):465–80, mar 1999. doi: 10.1016/S0006-2952(98)00268-8.

[4] Open Systems Pharmacology Suite Community. Open Systems Pharmacology Suite Manual, 2018. URL https://docs.open-systems-pharmacology.org/.

[5] M. Nishimura, H. Yaguti, H. Yoshitsugu, S. Naito, and T. Satoh. Tissue distribution of mRNA expression of human cytochrome P450 isoforms assessed by high-sensitivity real-time reverse transcription PCR. *Journal of the Pharmaceutical Society of Japan*, 123(5):369–75, may 2003.

[6] K. Rowland Yeo, R. L. Walsky, M. Jamei, A. Rostami-Hodjegan, and G. T. Tucker. Prediction of time-dependent CYP3A4 drug-drug interactions by physiologically based pharmacokinetic modelling: Impact of inactivation parameters and enzyme turnover. *European Journal of Pharmaceutical Sciences*, 43(3):160–73, jun 2011. doi: 10.1016/j.ejps.2011.04.008.

[7] D. J. Greenblatt, L. L. Von Moltke, J. S. Harmatz, G. Chen, J. L. Weemhoff, C. Jen, C. J. Kelley, B. W. LeDuc, and M. A. Zinny. Time course of recovery of cytochrome P450 3A function after single doses of grapefruit juice. *Clinical Pharmacology and Therapeutics*, 74(2):121–29, aug 2003. doi: 10.1016/S0009-9236(03)00118-8.

[8] B. Achour, A. Dantonio, M. Niosi, J. J. Novak, J. K. Fallon, J. Barber, P. C. Smith, A. Rostami-Hodjegan, and T. C. Goosen. Quantitative characterization of major hepatic UDP-glucuronosyltransferase enzymes in human liver microsomes: Comparison of two proteomic methods and correlation with catalytic activity. *Drug metabolism and disposition: the biological fate of chemicals*, 45(10):1102–112, 2017.

[9] B. Prasad, R. Evers, A. Gupta, C. E. C. A. Hop, L. Salphati, S. Shukla, S. V. Ambudkar, and J. D. Unadkat. Interindividual variability in hepatic organic anion - transporting polypeptides and P-glycoprotein (ABCB1) protein expression: quantification by liquid chromatography tandem mass spectroscopy and influence of genotype, age, and sex. *Drug metabolism and disposition: the biological fate of chemicals*, 42(1):78–88, 2014. doi: 10.1124/dmd.113.053819.

[10] M. Nishimura and S. Naito. Tissue-specific mRNA expression profiles of human ATP-binding cassette and solute carrier transporter superfamilies. *Drug metabolism and pharmacokinetics*, 20(6):452–77, 2005.

[11] D. Scotcher, S. Billington, J. Brown, C. R. Jones, C. D. A. Brown, A. Rostami-Hodjegan, and A. Galetin. Microsomal and cytosolic scaling factors in dog and human kidney cortex and application for in vitro-in vivo extrapolation of renal metabolic clearance. *Drug Metabolism and Disposition*, 45(5):556–568, 2017.

[12] R. C. Heel, R. N. Brogden, A. Carmine, P. A. Morley, T. M. Speight, and G. S. Avery. Ketoconazole: A Review of its Therapeutic Efficacy in Superficial and Systemic Fungal Infections. *Drugs*, 23(1):1–36, 1982. doi: 10.2165/00003495-198223010-00001.

[13] Y. Huang, J. L. Colaizzi, R. H. Bierman, R. Woestenborghs, and J. Heykants. Pharmacokinetics and dose proportionality of ketoconazole in normal volunteers. *Antimicrobial agents and chemotherapy*, 30(2):206–10, aug 1986. doi: 10.1002/j.1552-4604.1986.tb02962.x.

[14] T. W. F. Chin, M. Loeb, and I. W. Fong. Effects of an acidic beverage (Coca-Cola) on absorption of ketoconazole. *Antimicrobial agents and chemotherapy*, 39(8):1671–5, aug 1995. doi: 10.1128/AAC.39.8.1671.

[15] T. K. Daneshmend, D. W. Warnock, M. D. Ene, E. M. Johnson, G. Parker, M. D. Richardson, and C. J.C. Roberts.

Multiple dose pharmacokinetics of ketoconazole and their effects on antipyrine kinetics in man. *Journal of Antimicrobial Chemotherapy*, 12(2):185–188, 1983. doi: 10.1093/jac/12.2.185.

[16] T. K. Daneshmend, D. W. Warnock, M. D. Ene, E. M. Johnson, M. R. Potten, M. D. Richardson, and P. J. Williamson. Influence of food on the pharmacokinetics of ketoconazole. *Antimicrobial agents and chemotherapy*, 25(1):1–3, jan 1984. doi: 10.1007/BF01061429.

[17] U.S. Food and Drug Administration. Bioequivalence - application number: 74-971, 1998.

[18] C. A. Knupp, D. C. Brater, J. Relue, and R. H. Barbhaiya. Pharmacokinetics of didanosine and ketoconazole after coadministration to patients seropositive for the human immunodeficiency virus. *Journal of clinical pharmacology*, 33(10):912–7, oct 1993.

[19] P. T. Männistö, R. Mäntylä, S. Nykänen, U. Lamminsivu, and P. Ottoila. Impairing effect of food on ketoconazole absorption. *Antimicrobial agents and chemotherapy*, 21(5):730–3, may 1982. doi: 10.1128/AAC.21.5.730.

[20] H. R Sadeghnia and M. Hassanzadeh-Khayyat. Bioequivalency Study of Two Formulations of Ketoconazole Tablet in Healthy Volunteers. *Iranian Journal of Pharmaceutical Sciences*, 1(4):209–215, 2005.

[21] W. D. S. Solomon, P. Senthamil Selvan, K. Yeeran Gowda, U. K. Mandal, and T. K. Pal. Evaluation of bioequivalence of two formulations containing 200 mg of ketoconazole. *Asian Journal of Chemistry*, 19(7):5365–5371, 2007. ISSN 09707077.

[22] J. W. M. Van Der Meer, J. J. Keuning, H. W. Scheijgrond, J. Heykants, J. Van Cutsem, and J. Brugmans. The influence of gastric acidity on the bio-availability of ketoconazole. *Journal of Antimicrobial Chemotherapy*, 6(4): 552–554, 1980. doi: 10.1093/jac/6.4.552.

[23] K. H. Yuen, J. W. Wong, N. Billa, W. P. Choy, and T. Julianto. Comparative bioavailability study of two ketoconazole tablet preparations. *The Medical journal of Malaysia*, 54(4):482–6, dec 1999.

[24] R. E. Polk, M. A. Crouch, D. S. Israel, A. Pastor, B. M. Sadler, G. E. Chittick, W. T. Symonds, W. Gouldin, and Y. Lou. Pharmacokinetic interaction between ketoconazole and amprenavir after single doses in healthy men. *Pharmacotherapy*, 19(12):1378–84, dec 1999. doi: 10.1592/phco.19.18.1378.30905.

[25] S. C. Piscitelli, T. F. Goss, J. H. Wilton, D. T. D'Andrea, H. Goldstein, and J. J. Schentag. Effects of ranitidine and sucralfate on ketoconazole bioavailability. *Antimicrobial Agents and Chemotherapy*, 35(9):1765–1771, sep 1991. doi: 10.1128/AAC.35.9.1765.

[26] S. Sriwiriyajan, W. Mahatthanatrakul, W. Ridtitid, and S. Jaruratanasirikul. Effect of efavirenz on the pharmacokinetics of ketoconazole in HIV-infected patients. *European Journal of Clinical Pharmacology*, 63(5):479–483, 2007. doi: 10.1007/s00228-007-0282-8.

[27] J. Weiss, K. I. Foerster, M. Weber, J. Burhenne, G. Mikus, T. Lehr, and W. E. Haefeli. Does the circulating ketoconazole metabolite N-deacetyl ketoconazole contribute to the drug-drug interaction potential of the parent compound? *European Journal of Pharmaceutical Sciences*, 169:106076, 2022. doi: 10.1016/j.ejps.2021.106076.

[28] M. J. Boyce, K. J. Baisley, and S. J. Warrington. Pharmacokinetic interaction between domperidone and ketoconazole leads to QT prolongation in healthy volunteers: a randomized, placebo-controlled, double-blind, crossover study. *British journal of clinical pharmacology*, 73(3):411–21, mar 2012. doi: 10.1111/j.1365-2125.2011.04093.x.

[29] C. G. Patel, L. Li, S. Girgis, D. M. Kornhauser, E. U. Frevert, and D. W. Boulton. Two-way pharmacokinetic interaction studies between saxagliptin and cytochrome P450 substrates or inhibitors: simvastatin, diltiazem extended-release, and ketoconazole. *Clinical pharmacology : advances and applications*, 3(1):13–25, 2011. doi: 10.2147/CPAA.S15227.

[30] Tiseo, Perdomo, and Friedhoff. Concurrent administration of donepezil HCl and ketoconazole: assessment of pharmacokinetic changes following single and multiple doses. *British Journal of Clinical Pharmacology*, 46(S1): 30–34, 2002. doi: 10.1046/j.1365-2125.1998.0460s1030.x.

[31] M. B. Wire, C. H. Ballow, J. Borland, M. J. Shelton, Y. Lou, G. Yuen, J. Lin, and E. W. Lewis. Fosamprenavir plus ritonavir increases plasma ketoconazole and ritonavir exposure, while amprenavir exposure remains unchanged. *Antimicrobial Agents and Chemotherapy*, 51(8):2982–2984, 2007. doi: 10.1128/AAC.00008-07.

[32] D. J. Greenblatt, C. E. Wright, L. L. Von Moltke, J. S. Harmatz, B. L. Ehrenberg, L. M. Harrel, K. Corbett, M. Counihan, S. Tobias, and R. I. Shader. Ketoconazole inhibition of triazolam and alprazolam clearance: Differential kinetic and dynamic consequences. *Clinical Pharmacology and Therapeutics*, 64(3):237–247, 1998. doi: 10.1016/S0009-9236(98)90172-2.

[33] P. C. Craven, J. R. Graybill, J. H. Jorgensen, W. E. Dismukes, and B. E. Levine. High-dose ketoconazole for treatment of fungal infections of the central nervous system. *Annals of internal medicine*, 98(2):160–7, feb 1983. doi: 10.7326/0003-4819-98-2-160.

[34] A. Dallmann. IVIC with the particle dissolution module implemented in OSP. Available online: https://github.com/AndreDlm/IVIVC-with-particle-dissolution-module-in-OSP (accessed on 30 June 2022).

[35] Chemicalize. Ketoconazole.

[36] F. Taneri, T. Güneri, Z. Aigner, and M. Kata. Improvement in the Physicochemical Properties of Ketoconazole through Complexation with Cyclodextrin Derivatives. *Journal of Inclusion Phenomena and Macrocyclic Chemistry*, (44):257–260, jul 2002. doi: https://doi.org/10.1023/A:1023013523416.

[37] H. S. Ghazal, A. M. Dyas, J. L. Ford, and G. A. Hutcheon. The impact of food components on the intrinsic dissolution rate of ketoconazole Authors. *Drug Development And Industrial Pharmacy*, 41(10):1647–54, 2015.

[38] Chemspider. Ketoconazole entry (accessed on 01 May 2021). URL https://www.chemspider.com/Chemical-Structure.401695.html.

[39] R. S. Fisher, E. Rock, and L. S. Malmud. Effects of meal composition on gallbladder and gastric emptying in man. *Digestive Diseases and Sciences*, 32(12):1337–1344, dec 1987. doi: 10.1007/BF01296658.

[40] L. M. Berezhkovskiy. Volume of Distribution at Steady State for a Linear Pharmacokinetic System with Peripheral Elimination. *Journal of Pharmaceutical Sciences*, 93(6):1628–1640, jun 2004. doi: 10.1002/jps.20073.

[41] T. Fukami, A. Iida, K. Konishi, and M. Nakajima. Human arylacetamide deacetylase hydrolyzes ketoconazole to trigger hepatocellular toxicity. *Biochemical Pharmacology*, 116:153–161, 2016. doi: 10.1016/j.bcp.2016.07.007.

[42] K. Bourcier, R. Hyland, S. Kempshall, R. Jones, J. Maximilien, N. Irvine, and B. Jones. Investigation into UDP-Glucuronosyltransferase (UGT) Enzyme Kinetics of Imidazole- and Triazole-Containing Antifungal Drugs in Human Liver Microsomes and Recombinant UGT Enzymes. *Drug Metabolism and Disposition*, 38(6):923–929, jun 2010. doi: 10.1124/dmd.109.030676.

[43] E. J. Elder, J. C. Evans, B. D. Scherzer, J. E. Hitt, G. B. Kupperblatt, S. A. Saghir, and D. A. Markham. Preparation, characterization, and scale-up of ketoconazole with enhanced dissolution and bioavailability. *Drug Development and Industrial Pharmacy*, 33(7):755–765, 2007. doi: 10.1080/03639040601031882.

[44] Chemicalize. N-deactylketoconazole entry (accessed on 01 May 2021), 2021.

[45] R. Kawai, M. Lemaire, J. L. Steimer, A. Bruelisauer, W. Niederberger, and M. Rowland. Physiologically based pharmacokinetic study on a cyclosporin derivative, SDZ IMM 125. *Journal of pharmacokinetics and biopharmaceutics*, 22(5):327–65, oct 1994.

[46] T. Rodgers and M. Rowland. Physiologically based pharmacokinetic modelling 2: predicting the tissue distribution of acids, very weak bases, neutrals and zwitterions. *Journal of pharmaceutical sciences*, 95(6):1238–57, jun 2006. doi: 10.1002/jps.20502.

[47] R. J. Rodriguez and D. Acosta. Metabolism of ketoconazole and deacetylated ketoconazole by rat hepatic microsomes and flavin-containing monooxygenases. *Drug metabolism and disposition: the biological fate of chemicals*, 25(6):772–7, jun 1997.

[48] Chemicalize. N-deacetyl-N-hydroxyketoconazole entry (accessed on 01 May 2021), 2022.

[49] T. K. Daneshmend, D. W. Warnock, A. Turner, and C. J.C. Roberts. Pharmacokinetics of ketoconazole in normal subjects. *Journal of Antimicrobial Chemotherapy*, 8(4):299–304, 1981. doi: 10.1093/jac/8.4.299.

[50] E. J. Guest, L. Aarons, J. B. Houston, A. Rostami-Hodjegan, and A. Galetin. Critique of the two-fold measure of prediction success for ratios: application for the assessment of drug-drug interactions. *Drug metabolism and disposition: the biological fate of chemicals*, 39(2):170–3, feb 2011. doi: 10.1124/dmd.110.036103.

[51] E. D. Kharasch, S. Vangveravong, N. Buck, A. London, T. Kim, J. Blood, and R. H. Mach. Concurrent assessment of hepatic and intestinal cytochrome P450 3A activities using deuterated alfentanil. *Clinical pharmacology and therapeutics*, 89(4):562–70, apr 2011. doi: 10.1038/clpt.2010.313.

[52] X. Boulenc, O. Nicolas, S. Hermabessière, I. Zobouyan, V. Martin, Y. Donazzolo, and doi = 10.1007/s13318-014-0235-4 file = :C /Users/Fatima Marok/AppData/Local/Mendeley Ltd./Mendeley Desktop/Downloaded/Boulenc et al. - 2016 - CYP3A4-based drug-drug interaction CYP3A4 substrates' pharmacokinetic properties and ketoconazole dose regime(2).pdf:pdf isbn = 2107-0180 (Electronic)0̊378-7966 (Linking) journal = European Journal of Drug Metabolism and Pharmacokinetics keywords = Alprazolam,CYP3A4,Drug-drug interaction,Ketoconazole,Midazolam,PBPK simulation number = 1 pages = 45–54 pmid = 25374256 title = CYP3A4-based drug-drug interaction: CYP3A4 substrates' pharmacokinetic properties and ketoconazole dose regimen effect volume = 41 year = 2016 Ollier, C.

[53] B. Halama, N. Hohmann, J. Burhenne, J. Weiss, G. Mikus, and W. E. Haefeli. A nanogram dose of the CYP3A probe substrate midazolam to evaluate drug interactions. *Clinical Pharmacology and Therapeutics*, 93(6):564–571, 2013. ISSN 00099236. doi: 10.1038/clpt.2013.27.

[54] C. B. Eap, T. Buclin, G. Cucchia, D. Zullino, E. Hustert, G. Bleiber, K. Golay, A. Aubert, P. Baumann, A. Telenti, and R. Kerb. Oral administration of a low dose of midazolam (75 μg) as an in vivo probe for CYP3A activity. *European Journal of Clinical Pharmacology*, 60(4):237–246, 2004. doi: 10.1007/s00228-004-0762-z.

[55] E. Chung, A. N. Nafziger, D. J. Kazierad, and J. S. Bertino. Comparison of midazolam and simvastatin as cytochrome P450 3A probes. *Clinical Pharmacology and Therapeutics*, 79(4):350–361, 2006. doi: 10.1016/j.clpt.2005.11.016.

[56] G. Krishna, A. Moton, L. Ma, I. Savant, M. Martinho, M. Seiberling, and J. McLeod. Effects of oral posaconazole on the pharmacokinetic properties of oral and intravenous midazolam: A phase I, randomized, open-label, crossover study in healthy volunteers. *Clinical Therapeutics*, 31(2):286–298, 2009. doi: 10.1016/j.clinthera.2009.02.022.

[57] S. A. Stoch, E. Friedman, A. Maes, K. Yee, Y. Xu, P. Larson, M. Fitzgerald, J. Chodakewitz, and J. A. Wagner. Effect of different durations of ketoconazole dosing on the single-dose pharmacokinetics of midazolam: Shortening the paradigm. *Journal of Clinical Pharmacology*, 49(4):398–406, 2009. doi: 10.1177/0091270008331133.

[58] S. M. Tsunoda, R. L. Velez, L. L. von Moltke, and D. J. Greenblatt. Differentiation of intestinal and hepatic cytochrome P450 3A activity with use of midazolam as an in vivo probe: effect of ketoconazole. *Clinical pharmacology and therapeutics*, 66(5):461–71, nov 1999. doi: 10.1016/S0009-9236(99)70009-3.

[59] B. Liu, H. K. Crewe, M. Ozdemir, K. Rowland Yeo, G. Tucker, and A. Rostami-Hodjegan. The absorption kinetics of ketoconazole plays a major role in explaining the reported variability in the level of interaction with midazolam: Interplay between formulation and inhibition of gut wall and liver metabolism. *Biopharmaceutics and drug disposition*, 38(3):260–270, apr 2017. doi: 10.1002/bdd.2058.

[60] K. T. Olkkola, J. T. Backman, and P. J. Neuvonen. Midazolam should be avoided in patients receiving the systemic antimycotics ketoconazole or itraconazole. *Clinical pharmacology and therapeutics*, 55(5):481–5, may 1994.

[61] Y. W. F. Lam, C. L. Alfaro, L. Ereshefsky, and M. Miller. Pharmacokinetic and pharmacodynamic interactions of oral midazolam with ketoconazole, fluoxetine, fluvoxamine, and nefazodone. *Journal of clinical pharmacology*, 43(11): 1274–82, nov 2003. doi: 10.1177/0091270003259216.

[62] A. Varhe, K. T. Olkkola, and P. J. Neuvonen. Oral triazolam is potentially hazardous to patients receiving systemic antimycotics ketoconazole or itraconazole. *PHARMACOKINETICS AND DRUG DISPOSITION*, pages 601–607, 1994.

[63] U. L. Larsen, Hyldahl L. Olesen, Guldborg C. Nyvold, J. Eriksen, P. Jakobsen, M. Østergaard, H. Autrup, and V. Andersen. Human intestinal P-glycoprotein activity estimated by the model substrate digoxin. *Scandinavian Journal of Clinical and Laboratory Investigation*, 67(2):123–134, 2007. doi: 10.1080/00365510600986084.

[64] N. Hanke, S. Frechen, D. Moj, H. Britz, T. Eissing, T. Wendl, and T. Lehr. PBPK models for CYP3A4 and P-gp

DDI prediction: a modeling network of rifampicin, itraconazole, clarithromycin, midazolam, alfentanil and digoxin - Supplementary document. *CPT: pharmacometrics & systems pharmacology*, aug 2018.

[65] A. Frechen, S.; Dallmann. Building and Evaluation of a PBPK Model for Alprazolam in Healthy Adults. Available online: https://github.com/Open-Systems-Pharmacology/Alprazolam-Model/releases/tag/v1.1 (accessed on 30 June 2022).

[66] J.; Solodenko, S.; Frechen, and A. Dallmann. Building and Evaluation of a PBPK Model for Triazolam in Healthy Adults. Available online: https://github.com/Open-Systems-Pharmacology/Triazolam-Model/releases/tag/v1.1 (accessed on 30 June 2022).
